# Crystal structure of the human 5-HT_1B_ serotonin receptor bound to an inverse agonist

**DOI:** 10.1038/s41421-018-0009-2

**Published:** 2018-03-13

**Authors:** Wanchao Yin, X. Edward Zhou, Dehua Yang, Parker W. de Waal, Meitian Wang, Antao Dai, Xiaoqing Cai, Chia-Ying Huang, Ping Liu, Xiaoxi Wang, Yanting Yin, Bo Liu, Yu Zhou, Jiang Wang, Hong Liu, Martin Caffrey, Karsten Melcher, Yechun Xu, Ming-Wei Wang, H. Eric Xu, Yi Jiang

**Affiliations:** 1 VARI-SIMM Center, Center for Structure and Function of Drug Targets, The CAS Key Laboratory of Receptor Research, Shanghai Institute of Materia Medica, Chinese Academy of Sciences, Shanghai, 201203 China; 2 University of Chinese Academy of Sciences, No.19 A Yuquan Road, Beijing, 100049 China; 3 The CAS Key Laboratory of Receptor Research, Shanghai Institute of Materia Medica, Chinese Academy of Sciences (CAS), Shanghai, 201203 China; 4 Laboratory of Structural Sciences, Van Andel Research Institute, Grand Rapids, MI 49503 USA; 5 The National Center for Drug Screening, Shanghai, 201203 China; 6 Swiss Light Source, Paul Scherrer Institute, Villigen, 5232 Switzerland; 7 State Key Laboratory of Drug Research, Shanghai Institute of Materia Medica, CAS, Shanghai, 201203 China; 8 Membrane Structural and Functional Biology Group, Schools of Medicine and Biochemistry and Immunology, Trinity College Dublin, Dublin, Ireland; 9 School of Pharmacy, Fudan University, Shanghai, 201203 China; 10 School of Life Science and Technology, ShanghaiTech University, Pudong, Shanghai 201203 China

## Abstract

5-hydroxytryptamine (5-HT, also known as serotonin) regulates many physiological processes through the 5-HT receptor family. Here we report the crystal structure of 5-HT_1B_ subtype receptor (5-HT_1B_R) bound to the psychotropic serotonin receptor inverse agonist methiothepin (MT). Crystallization was facilitated by replacing ICL3 with a novel optimized variant of BRIL (OB1) that enhances the formation of intermolecular polar interactions, making OB1 a potential useful tool for structural studies of membrane proteins. Unlike the agonist ergotamine (ERG), MT occupies only the conserved orthosteric binding pocket, explaining the wide spectrum effect of MT on serotonin receptors. Compared with ERG, MT shifts toward TM6 and sterically pushes residues W327^6.48^, F330^6.50^ and F331^6.51^ from inside the orthosteric binding pocket, leading to an outward movement of the extracellular end and a corresponding inward shift of the intracellular end of TM6, a feature shared by other reported inactive G protein-coupled receptor (GPCR) structures. Together with the previous agonist-bound serotonin receptor structures, the inverse agonist-bound 5-HT_1B_R structure identifies a basis for the ligand-mediated switch of 5-HT_1B_R activity and provides a structural understanding of the inactivation mechanism of 5-HT_1B_R and some other class A GPCRs, characterized by ligand-induced outward movement of the extracellular end of TM6 that is coupled with inward movement of the cytoplasmic end of this helix.

## Introduction

G-protein-coupled receptors (GPCRs) are targets of more than one-third of the currently used therapeutic agents, and comprise the largest membrane protein family. GPCRs sense signaling molecules outside of the cells and activate multiple intracellular signaling pathways through conformational changes in the cytoplasmic side of the transmembrane domain (TMD). Recent progresses made in the structural and functional studies of the GPCR superfamily provide unprecedented insights into molecular mechanisms of GPCR signal transduction. Unfortunately, GPCR crystallization remains difficult due to their low expression levels, instability during purification, and limited polar surface for protein–protein packing interactions in the aqueous phase that are required for crystallization of membrane proteins. These technical hurdles have been partly overcome by the use of GPCR fusion partners, which have greatly accelerated GPCR structural studies over the past decade by aiding in protein expression, purification, and crystallization^1^. The Protein Data Bank lists various fusion partners, including T4 lysozyme (T4L)^2, 3^ “disulfide-stabilized T4L” (dsT4L)^4^, “minimal T4L” (mT4L)^4^, thermostabilized apocytochrome b562 RIL (BRIL)^5–7^, flavodoxin^8^, rubredoxin^9^, and *Pyrococcus abysii* glycogen synthase (PGS)^10^, that have facilitated GPCR crystallization. Interactions between fusion partners or between fusion partner and the GPCR can help overcome the disadvantage of the minimal polar surface area of GPCRs for protein–protein packing interactions in aqueous phase, thus improving crystallizability of GPCR fusion proteins. Since none of these fusion partners provides a universal solution for GPCR crystallization, designing new fusion partners or engineering currently available ones represents an effective strategy for GPCR crystallization and crystal optimization. Notably, mT4L and dsT4L, designed to optimize crystal quality by providing alternative packing interactions, were successfully utilized in structure determination of the M3 muscarinic receptor^4^. Here we present a modified BRIL-based fusion partner OB1 (optimization variant 1 of BRIL), which significantly improved the crystallizability of the 5-HT_1B_R-fusion protein. Using OB1 as a fusion partner, we determined the crystal structure of 5-HT_1B_R bound to an inverse agonist, methiothepin (MT)^11–14^.

The serotonergic system regulates a wide range of human physiological processes^15^, including modulation of smooth muscle contraction, platelet aggregation, mood, wakefulness, anxiety, and perception through activation of 5-HT receptors by the neuromodulator serotonin (5-hydroxytryptamine or 5-HT). With the exception of the ion channel 5-HT_3_R subfamily, 5-HT receptors consist of 13 GPCRs that are grouped into six subclasses^16^. The serotonergic system is one of the most important targets for many therapeutics agents, including antimigraine mediations, antidepressants, antipsychotics, anxiolytics, and anti-obesity drugs^17^. The wide distribution and functional diversity of 5-HT receptors explain the diverse side-effects of these agents targeting this receptor family, thus making drug discovery extremely challenging. Several serotonergic drugs were withdrawn because of unexpected adverse properties that resulted from their off-target actions^18–20^.

5-HT_1B_R is primarily expressed in presynaptic neurons. Upon 5-HT binding, 5-HT_1B_R couples to Gi or Go proteins to reduce the release of serotonin into the synaptic cleft^21, 22^. Conversely, selective 5-HT_1B_R antagonists specifically increase the level of serotonin in the synaptic cleft and serve as potential antidepressant agents. On the other hand, 5-HT_1B_R agonists, including ERG and dihydroergotamine (DHE), have been widely used clinically for their antimigraine effect^23, 24^. The crystal structures of the human 5-HT_1B_R bound to ERG and DHE have been reported^6, 7^. Together with the crystal structure of the human 5-HT_2B_R bound to ERG^6, 7^, these structures provide detailed information for understanding the molecular recognition and functional selectivity of serotonin agonists by the receptors. To date, there is no structure of any antagonist-bound or inverse agonist-bound 5-HT receptor to provide structural information that is indispensable for understanding the molecular recognition of inverse agonist or antagonist by 5-HT receptors, and for structure-based drug discovery (SBDD) of more effective and more specific antagonists or inverse agonists as therapeutic agents targeting the serotonergic system^25^. Here we report the first structure of 5-HT_1B_R, a member of the serotonin receptor family, bound to its inverse agonist MT. The structure reveals the basis of ligand-induced repression of 5-HT_1B_R activity and provides a structural understanding of the inactivation mechanism of 5-HT_1B_R and other class A GPCRs.

## Results and discussion

### Engineering a BRIL fusion partner to facilitate 5-HT_1B_R crystallization

**Fig. 1:**
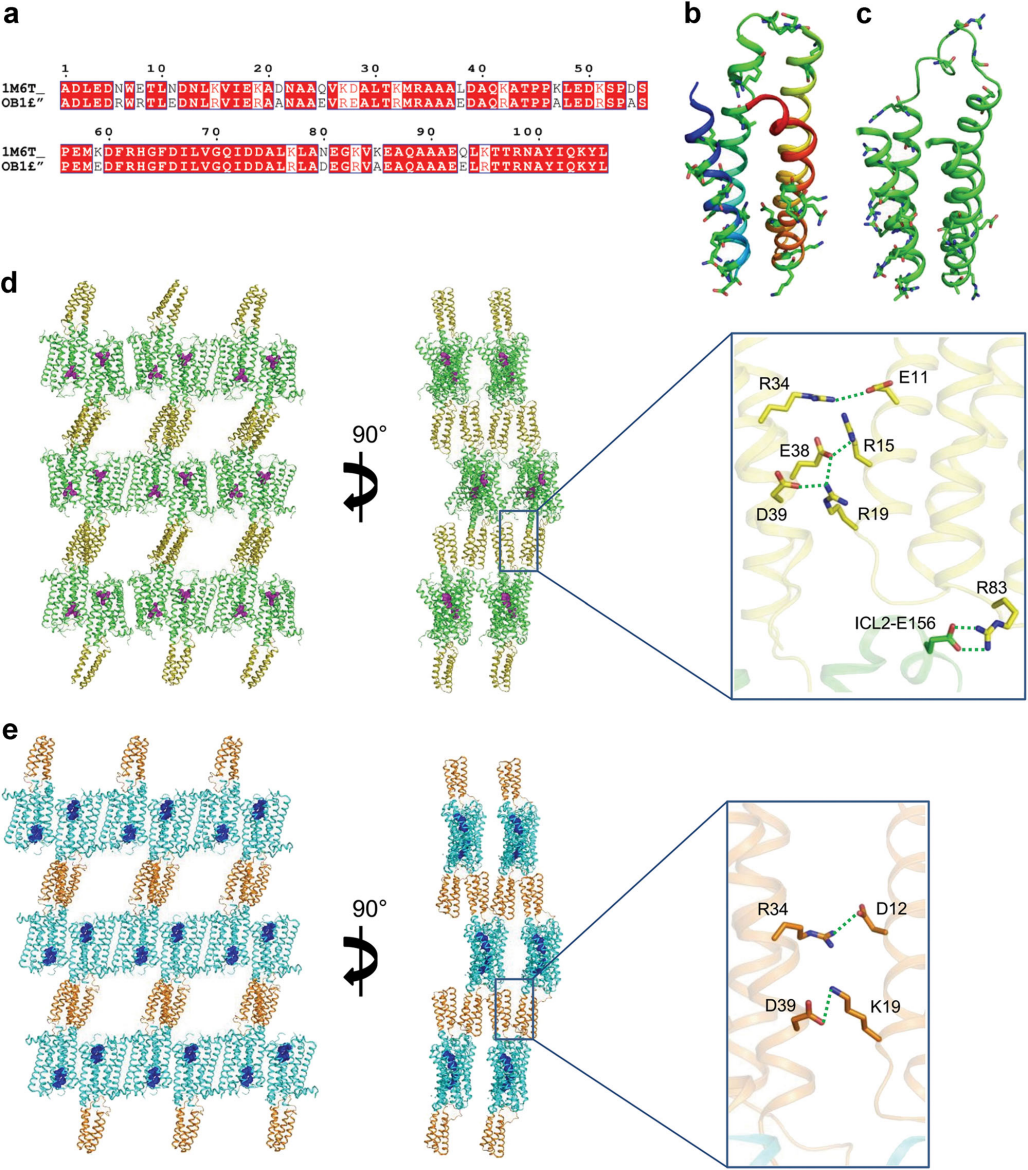
The fusion partner OB1 facilitates crystallization of the 5-HT_1B_R- inverse agonist MT complex. **a** Sequence alignment of BRIL and OB1 showing the mutations introduced into BRIL (PDB code: 1M6T) to improve crystallization of fusion proteins. **b** BRIL structural model shown in rainbow colors (N terminus, blue; C terminus, red) with mutated residues displayed as sticks. **c** Structural model of the OB1 fusion protein in the structure of 5-HT_1B_R/MT complex, with mutated residues shown as sticks. **d** Two crystal packing views of the 5-HT_1B_R/MT complex along cell axes b (left) and c (right) in the space group C2. Details of the polar interactions between neighboring symmetry OB1 molecules are shown in the box on the right. 5-HT_1B_R is in green, BRIL fusion partner in orange-olive, and the ligand MT in magenta. The interactions between interface residues of the neighboring OB1 molecules can be defined by electron density or by computational modeling for those whose side chains lack clear electron density. **e** Two views of crystal packing of the 5-HT_1B_R/ERG complex in the space group C2 with the detailed polar interactions mediated by BRIL shown in the box on the right. 5-HT_1B_R is shown in cyan, BRIL fusion protein in orange and the ligand ERG in blue

The 5-HT_1B_R in complex with the inverse agonist MT was crystallized in lipid cubic phase (LCP) with monoolein as a host lipid. To facilitate crystallization, we replaced the third intracellular loop (ICL3) of 5-HT_1B_R with BRIL, which is the same strategy used in structure determination of 5-HT_1B_R/ERG complex^6, 7^. Although we obtained small crystals with a number of inverse agonists or antagonists, the diffraction quality could not be improved beyond 7 Å despite extensive optimization of various crystallization conditions. Since BRIL contributed to most of the polar packing interactions in the 5-HT_1B_R/ERG complex crystals, we focused on optimization of the sequence of BRIL, hoping to improve the crystal diffraction quality. It has been reported that the success of membrane protein crystallization in LCP is highly dependent on the potential of target proteins to form specific intermolecular interactions in the aqueous phase to help crystal packing contacts^26^. Certain residues with large and flexible side chains, such as Lys, Gln, and Glu, are thought to interfere with the proteins to form stable crystal packing interactions^27–30^. We therefore introduced 22 mutations at Lys, Gln, and Glu residues in BRIL to create an “optimized variant 1 of BRIL (OB1)”, for the purpose of reducing surface entropy and strengthening specific polar interactions within BRIL and/or between BRIL fusions of the neighboring symmetry molecules (Fig. 1a–c). The 5-HT_1B_R fusion with OB1 retains the same ligand binding affinity as the wild type receptor (Table S1). As expected, OB1-fused 5-HT_1B_R was readily crystallized in many more crystallization conditions than the BRIL-fused receptor. For example, from the OB1 fusion protein, crystals appeared in 15 conditions with 10 different salts, while crystals were only seen in five conditions with four different salts from BRIL-fused receptor, when StockOptions™ salt kit from Hampton Research was used. With the aid of OB1, we obtained a crystal that diffracted to 3.90 Å resolution. The structure was solved by molecular replacement with the 5-HT_1B_R/ERG complex as the initial model and the final structure was refined using DEN^31^ and ROSETTA^32^ computational methods to an R-factor of 27.4% and a free-R of 28.9% (Table S2 and Figure S1) with excellent geometry and Ramachadran statistics, and a Molprobity score of 1.3 (Table S2).

### Crystal packing interactions and the effect of OB1 on crystallization of 5-HT_1B_R

The 22 mutations in BRIL (PDB code 1M6T^33^) can be classified into two different groups. Among the first group are mutations D21A, K47A, D54A, and K85A that were designed based on the theory of the surface entropy reduction^27^. The second group includes mutations E8R, N11E, K15R, K19R, Q25E, K32R, L38E, and K83R, which were introduced to strengthen specific inter-molecular or intra-molecular polar interactions. While the resolution of the structure limits the direct observation of small or flexible side chains, the rotamers of many large residues including the polar or charged residues involved in intramolecular or intermolecular electrostatic interactions, can be unambiguously defined by the density with additional geometric constraints (Fig. 1 and S2).

In the structure, the newly introduced charged residues were observed to form salt bridges that are involved in the charge interaction network at the interfaces between the fused OB1 of symmetry molecules. R8, introduced by E8R mutation, forms charge interaction with D12 on helix 1 (Figures S2A, residue numbers are based on the sequence of BRIL). E11 from mutation N11E directly interacts with R98 of helix 4 (Figure S2B). All above ion-pairs are parts of a charge interaction network formed by residues E11, D12, R15, and R19 of helix 1, and R98 of helix 4, of one molecule (A or B), with residues R34, E38, and D39 (helix 1) from the adjacent symmetry molecule (A or B) (Fig. 1d right panel). Mutation of lysine to arginine did not change the charge of the residues, but could strengthen electrostatic interaction between ion-pair residues due to the increased rigidity and the multi-point binding ability of the arginine side chain. Examples are mutations K15R and K19R, which introduced arginine residues that strengthened the multi-residue charge interaction networks (Fig. 1d right panel). In addition, several alanine residues introduced by substitution of charged residues were found to be involved in non-polar intramolecular (K47A) or intermolecular (D21A, K85A) interfaces, indicating their potential contributions to the crystal packing interactions. The alanine from D54A substitution was found at the solvent exposed surface, which reduced surface entropy of the protein, and likely benefited crystal growth.

The high efficiency of OB1 that improved 5-HT_1B_R crystallization was due to the enhanced packing arrangement facilitated by the charge interaction network between adjacent OB1 fusions of the complex molecules in the aqueous phase of the crystals.

There are two complex molecules in the asymmetric unit of the 5-HT_1B_R/MT crystals but only one complex was seen in the 5-HT_1B_R/ERG crystals. Both crystals of the MT-bound and ERG-bound complexes are canonical type 1 membrane protein crystals with alternating layers of BRIL or OB1 (aqueous layer) and 5-HT_1B_R (lipid layer), and their crystal packing is largely mediated by BRIL-BRIL or OB1-OB1 interactions, respectively (Fig. 1d, e). For the BRIL-BRIL packing arrangement in the 5-HT_1B_R/ERG crystals, there were only two intermolecular salt bridges, mediated by D12 and R34, and K19 and D39, respectively (Fig. 1e). In contrast, the OB1 packing arrangement is maintained by multi-residue charge interaction networks, widely distributed between helices of the same OB1 fusion and those of the symmetry molecules. The charge-introducing mutations provide charged residues for the formation of the intermolecular charge network. In addition to those involved in the charge interaction network between OB1 fusions, R83 in OB1 from mutation K83R forms a strong salt bridge with E156 in ICL2 of the adjacent symmetry molecule which provides an additional crystal packing interaction that contributes to the crystal formation.

### Overall structure of the 5-HT_1B_R/MT complex

**Fig. 2:**
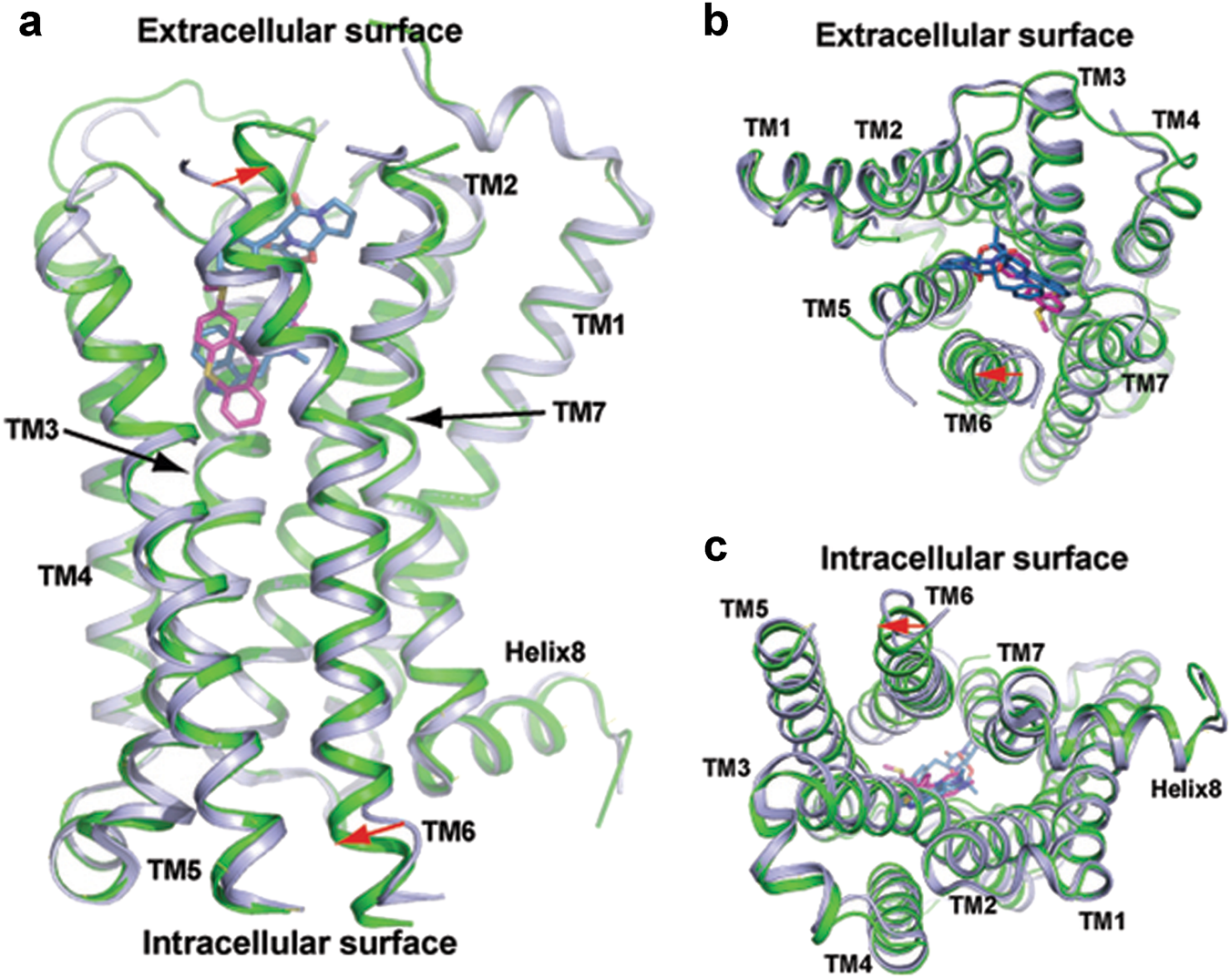
5-HT_1B_R/MT complex crystal structure and its comparison with 5-HT_1B_R/ERG complex. **a** Superposition of 5-HT_1B_R/MT with 5-HT_1B_R/ERG structures (PDB code: 4IAR), a side view, **b** an extracellular view, and **c** an intracellular view. 5-HT_1B_R/MT complex is shown in green/magenta and 5-HT_1B_R/ERG in light blue/dark blue. The red arrow indicates the movement of TM6 in the 5-HT_1B_R/MT structure compared to that of 5-HT_1B_R/ERG

**Fig. 3:**
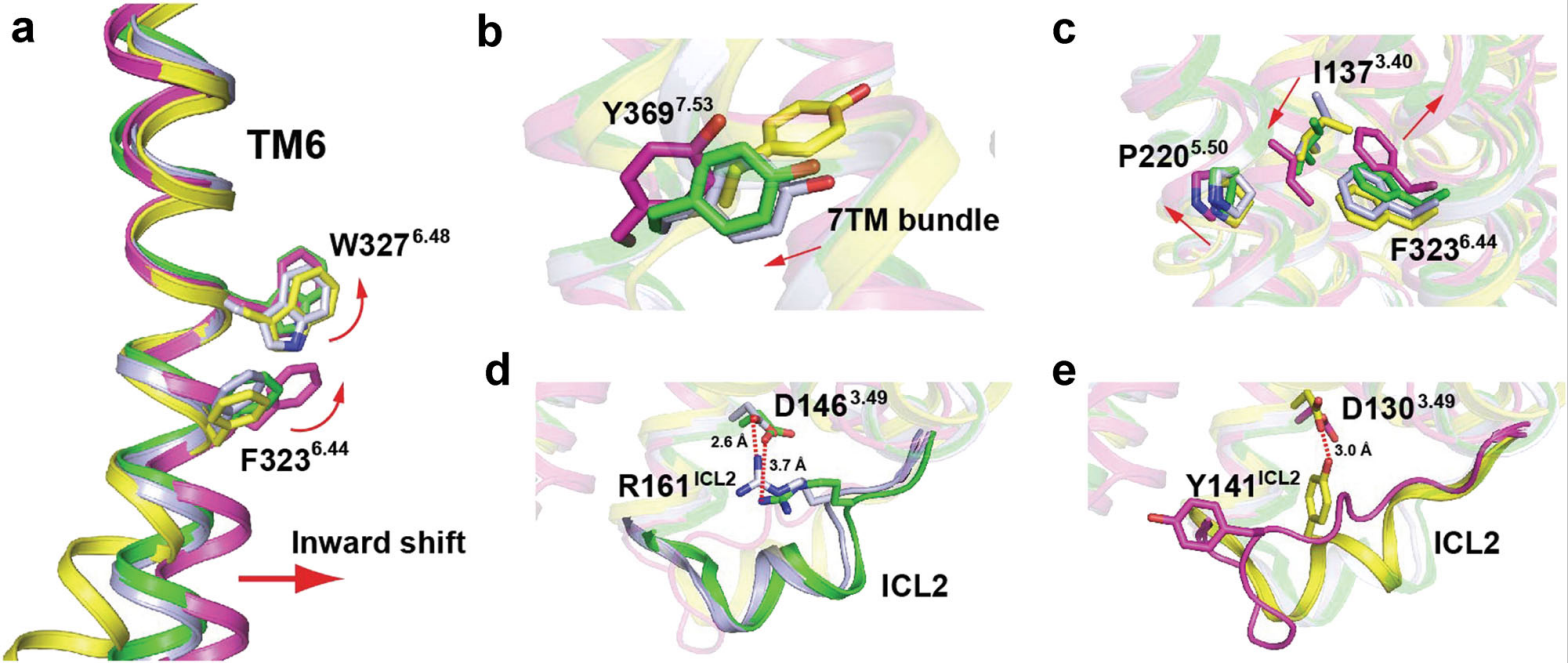
The 5-HT_1B_R/MT structure represents an intermediate state between active and inactive conformations. **a** The conformation of TM6 of the 5-HT_1B_R/MT complex (green) is compared with those of 5-HT_1B_R/ERG (PDB code: 4IAR, light blue), β_2_-AR/carazolol (inverse agonist, PDB code: 2RH1, magenta), and β_2_-AR/BI-167107 (agonist, PDB code: 3SN6, yellow). **b**, **c** Shifts of conserved residues in “micro switches” indicates that the 5-HT_1B_R/MT complex is largely in an inactive conformation. **b** “NPxxY”; and **c** “P-I-F”. Amino acid number labeling is based on the sequence of 5-HT_1B_R. Red arrows indicate the displacements of TM6 and its residues in inactive conformations compared with those in active ones. Side chains of conserved residues are shown as sticks. **d**, **e** Side chains of residues in micro switch “DRY”. D146^3.19^ can form salt bridges within R161^ICL2^ in both 5-HT_1B_R/MT and 5-HT_1B_R/ERG structures (**d**), while a similar polar interaction can only be observed in β_2_-AR/BI-167107 structure between D130^3.49^ and Y141^ICL2^ (**e**)

The overall architecture of the 5-HT_1B_/MT structure consists of a canonical bundle of seven transmembrane helices and is similar to that of 5-HT_1B_R/ERG with an overall RMSD of 1.1 Å between the backbone Cα atoms of the two receptor complexes. Compared to the agonist-bound structure, the most striking conformational difference in the MT-bound structure is the outward shift of the extracellular end of TM6 and a corresponding inward movement in the intracellular side of TM6 (red arrows in Fig. 2). A hallmark of GPCR activation is the outward movement of the cytoplasmic end of TM6, which expands the cytoplasmic pocket of the TM bundle for coupling downstream signaling effectors such as G proteins and arrestins^33–35^. Conversely, the inactive GPCR structures display an inwardly positioned cytoplasmic side of TM6, which closes the cytoplasmic pocket to prevent the coupling of the receptor with downstream effectors. The inward movement of TM6 at the cytoplasmic side shown in our structure is thus consistent with the inverse agonist property of MT, although the shift is smaller in magnitude than that seen in the inverse agonist bound β_2_-AR structure (Fig. 3a). The smaller inward shift of the cytoplasmic side of TM6 in our structure is likely due to the constraint by OB1, which was fused between TM5 and TM6 without additional linker residues, thereby limiting the freedom for inward movement of the intracellular side of TM6. Thus the 5-HT_1B_R/MT structure with the OB1 fusion may represent an intermediate state that is prone to transition to an inactive conformation, but is locked by the OB1 fusion.

The transition state of the 5-HT_1B_R/MT structure between active and inactive conformations is further supported by molecular dynamics (MD) simulations. Significant structural deformation can be observed at the linking region between 5-HT_1B_R and OB1 fusion partner in as little as 70 ns MD, suggesting that OB1 places considerable steric strains on receptor and prevents the receptor from being inactivated (Figure S3A). The data from four independent 500 ns MD simulations of the 5-HT_1B_R/MT structure with OB1 removed from the fusion indicate that removal of OB1 allows for a significantly decrease of cytoplasmic pocket volume (Figure S3B) and an additional 6 Å inward shift of TM6 at the cytoplasmic end (Figures S3D and S3E), resulting in an increased similarity to other inactive GPCR structures bound with inverse agonists. Furthermore, an additional 3 Å outward shift was also observed at the extracellular end of TM6 (Figure S3D), probably because the removal of the fused OB1 from the fusion receptor releases the constraints to the whole TM6 and allows the extracellular side of TM6 to adopt its natural MT-bound conformation. To this extent, coordinates for simulation snapshots have been included as supplemental files.

In addition to the conformation of TM6, several highly conserved residues known as “micro switches” that are responsible for helical movements can serve as structural indicators for GPCR activation and inactivation^36, 37^. The side chains of these “micro switch” residues in the 5-HT_1B_R/MT structure display positions or rotamers comparable to those in inactive GPCR structures, including (i) the rotamers of W327^6.48^ and F323^6.44^ in the “CWxP” motif in TM6 (Fig. 3a); (ii) a movement of Y369^7.53^ (“NPxxY”) away from the 7TM bundle (Fig. 3b); and (iii) the outward shift of P220^5.50^, a rotamer switch of I137^3.40^, and movement of the F323^6.44^ side chain of the “P-I-F” motif (Fig. 3c). Furthermore, the conserved structure rearrangements of residues I^3.46^, L^6.37^, and Y^7.53^ of 5-HT_1B_R/MT compared to those of 5-HT_1B_R/ERG are consistent with the model of convergence of GPCR activation pathways (Figure S4)^38^. This convergence is mediated by a strikingly conserved rearrangement of residues in helices 3, 6, and 7. The salt bridge between D146^3.49^ and R147^3.50^ in the DRY motif, a key feature of inactive GPCR conformation seems unclear in the 5-HT_1B_R/MT structure. Residue D146^3.49^, however, forms an additional salt bridge with R161 on ICL2, which may weaken the interaction network among the residues of this “DRY” motif (Fig. 3d). In the four independent 500 ns MD simulations, formation of the DRY motif was observed only once that accompany the unfolding of ICL2 helix, leading to a strong salt bridge formation between D146^3.49^ and R147^3.50^ in the “DRY” motif (Figure S5). Together, these analyses of the 5-HT_1B_R/MT structure and our MD results indicate that the 5-HT_1B_R/MT structure is in an intermediate state between the active and inactive conformations, which is prone to adopt an inactive conformation when the fusion OB1 is removed, consistent with the inverse agonist property of MT.

### The structure of MT in the 5-HT_1B_R ligand binding pocket

**Fig. 4:**
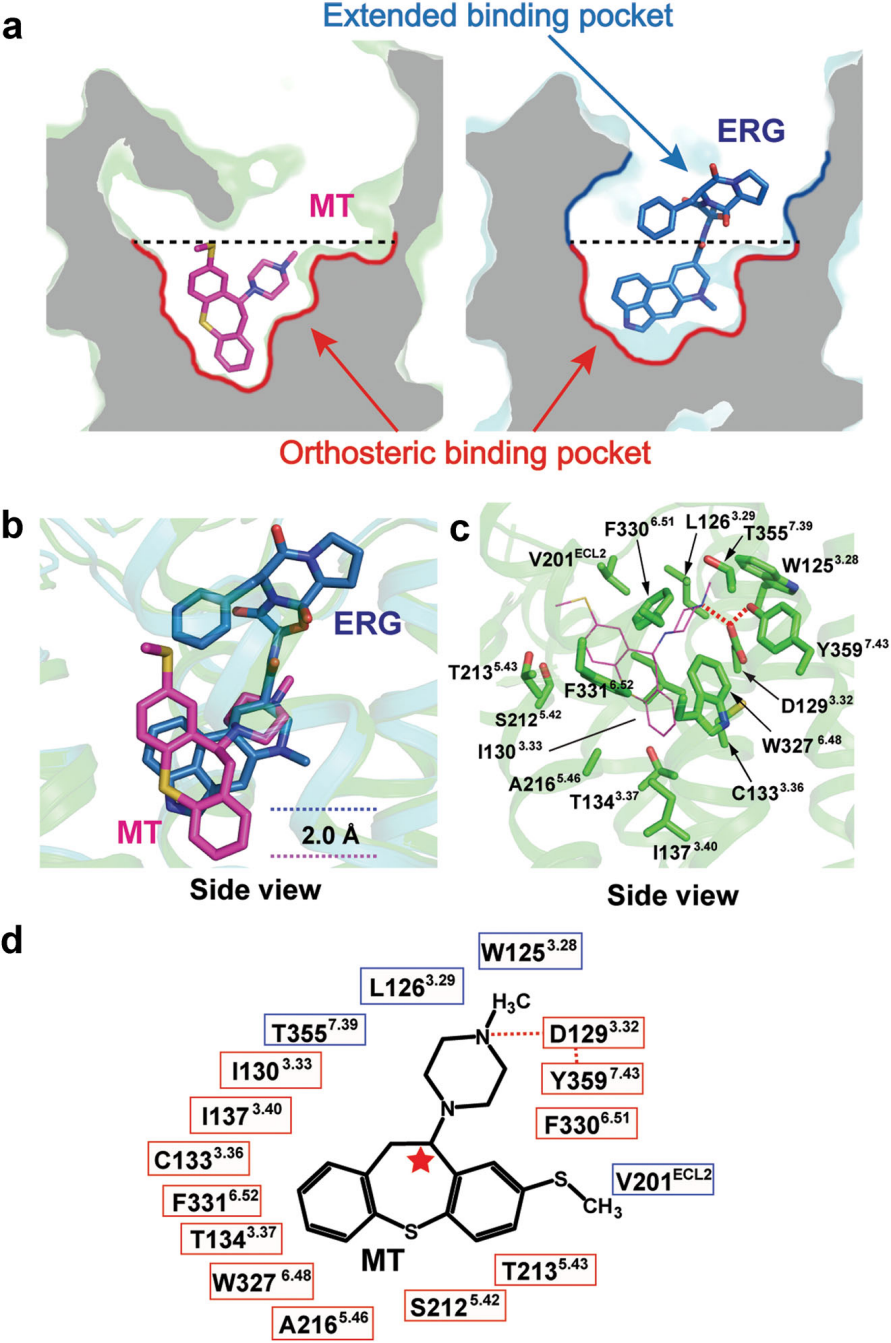
Ligand binding pockets of 5-HT_1B_R in complex with MT and ERG. **a** Overall presentation of 5-HT_1B_R ligand binding pockets in complex with MT (left) and ERG (right). The orthosteric and extended binding pockets are shown in red and blue, respectively. **b** The superposition of MT (magenta) and ERG (blue) in the 5-HT_1B_R ligand binding pockets. MT lies in the pocket 2.0 Å deeper than ERG. **c** MT in the ligand binding pocket with surrounding receptor residues. MT is shown as magenta line model. Residues in the pocket are shown as green sticks. **d** Schematic 2D presentation of interactions between 5-HT_1B_R and MT. Residues in the orthosteric pocket are shown in red boxes, and residues in the extended binding pocket are presented in blue boxes. Polar interactions are shown as red dashed lines. The chiral carbon atom within the MT S-isomer is labeled with a red star

The ligand MT used in crystallization is a 1:1 mixture of R/S-isomers with a chiral carbon atom (red star in Fig. 4d) connected to the piperazine ring. The structure reveals that the S-isomer fits the electron density better than the R-isomer (Figure S6). The 3-D feature of the 5-HT_1B_R ligand binding pocket displays a better fit for binding the S-isomer with a reasonable ligand–protein interface (Fig. 4a). In addition, the binding interface of the MT S-isomer remained stable for all 500 ns simulations while the R-isomer resulted in significant deformation of the ligand binding pocket and displacement of the ligand, further supporting the observed binding mode of MT in the 5-HT_1B_R ligand binding pocket. As previously noted, removal of OB1 relaxes strain on the TMD bundle and allows the extracellular portion of TM6 to move outwardly by approximately 3 Å. In this relaxed mode, MT moves away from the crystallographic to reach upwards of 1 Å deeper into the orthosteric binding pocket (Figures S7C, D). The dihydrodibenzo thiepine moiety further adopts a minimal energy conformation, in agreement with its predicted optimal geometry by the density functional theory (DFT; Figures S7A, B), where the methylsulfanyl group pushes on TM6 to facilitate receptor inactivation (Figure S7E).

It was previously reported that the ligand binding pocket of 5-HT_1B_R comprises a lower orthosteric pocket that contributes the ligand binding affinity and an upper extended binding pocket that determines ligand binding selectivity^6, 7^ (Fig. 4a). Unlike ERG, which occupies both lower and upper pockets, MT is located deeply in the TMD core and occupies the lower orthosteric pocket with few interactions with residues in the extended binding pocket (Fig. 4a, b). Because the residues comprising the orthosteric pocket are highly conserved, the absence of interactions with residues in the extended upper binding pocket provides a structural explanation for the low selectivity of MT for members of the serotonin receptor family^39–43^ (Figures S8 and S9). Notably, there is a salt bridge between the tertiary amine moiety in the piperazine ring of MT and the side chain of D129^3.32^ of 5-HT_1B_R. Together with Y359^7.43^, D129^3.32^ forms a polar interaction network anchoring MT in the largely hydrophobic binding pocket (Fig. 4c, d). Mutation of the highly conserved residue D^3.32^ to Ala abolished ligand-binding of both agonist and antagonist to 5-HT_1B_R (Table S3). Interestingly, D^3.32^ also forms a salt bridge with ergolines from ERG^7^ and LSD^44^ (Figure S10) in ligand-bound 5-HT receptor structures, an interaction strictly conserved in aminergic receptors^45^, highlighting the significance of D^3.32^ for ligand binding affinity to 5-HT receptors and other aminergic receptors.

Similar to other members of class A GPCR family, the 5-HT_1B_R ligand binding pocket is composed of residues from TM3, TM5, TM6, and TM7^46^. MT is inserted deeper into the hydrophobic orthosteric pocket by as much as 2.0 Å compared to ERG-bound 5-HT_1B_ receptor (Fig. 4b), and 4.0 Å compared to LSD-bound 5-HT_2B_ receptor (Figure S10A), indicating a deeper binding mode of inverse agonist to the helix core of 5-HT_1B_R than that of an agonist to the receptor. Surrounding the dihydrodibenzo thiepine moiety of MT are mostly hydrophobic residues, including C133^3.36^, T134^3.37^, I137^3.40^, T213^5.43^, A216^5.46^, W327^6.48^, F330^6.51^, F331^6.52^, and T355^7.39^, which form a broader hydrophobic pocket to accommodate the triple-ring moiety of MT (Fig. 4c, d). Most notably, residues Y359^7.43^, W327^6.48^, F331^6.52^, and F330^6.51^ formed an aromatic cage that encloses the ligand (Fig. 4c), and alanine mutations of any of these four residues totally abolished ligand binding (Table S3), indicating their crucial role in 5-HT_1B_R ligand binding. Alanine mutations of other hydrophobic residues that contact the bound ligand also resulted in reduced ligand binding affinity, in agreement with the observed ligand-receptor binding mode displayed in the complex structure.

The structure of the 5-HT_1B_R binding pocket can guide the discovery of more potent and highly selective ligands for 5-HT_1B_ receptor. First, a benzene-like ring group which can reach the bottom of the orthosteric binding pocket as deeply as MT may be required to provide antagonist or inverse agonist activity of the ligand. Second, a positively charged amine group or other polar group that interacts with the highly conserved residue D129^3.32^, as well as a hydrophobic group embraced by aromatic residues of the receptor, are determinants for ligand binding potency. Finally, more polar groups of the ligand interacted with polar residues in TM3 of the receptor, as exemplified by the indole N–H hydrogen of ERG and T134^3.37^ of 5-HT_1B_R (Figure S10C)^7^, which could help draw the ligand close to TM3, a structural and functional hub of the receptor^46^. Additionally, because the 5-HT_1B_R/MT complex structure lacks ligand occupancy in the upper extended binding pocket, more crystal structures in complex with bulkier antagonist or inverse agonist ligands could reveal more interaction information between the ligands and residues in the extended binding pocket, which are needed to clarify selectivity of inverse agonists/antagonists against different 5-HT receptors.

### A putative inactivation mechanism for 5-HT_1B_R and other class A GPCRs with known structures

**Fig. 5:**
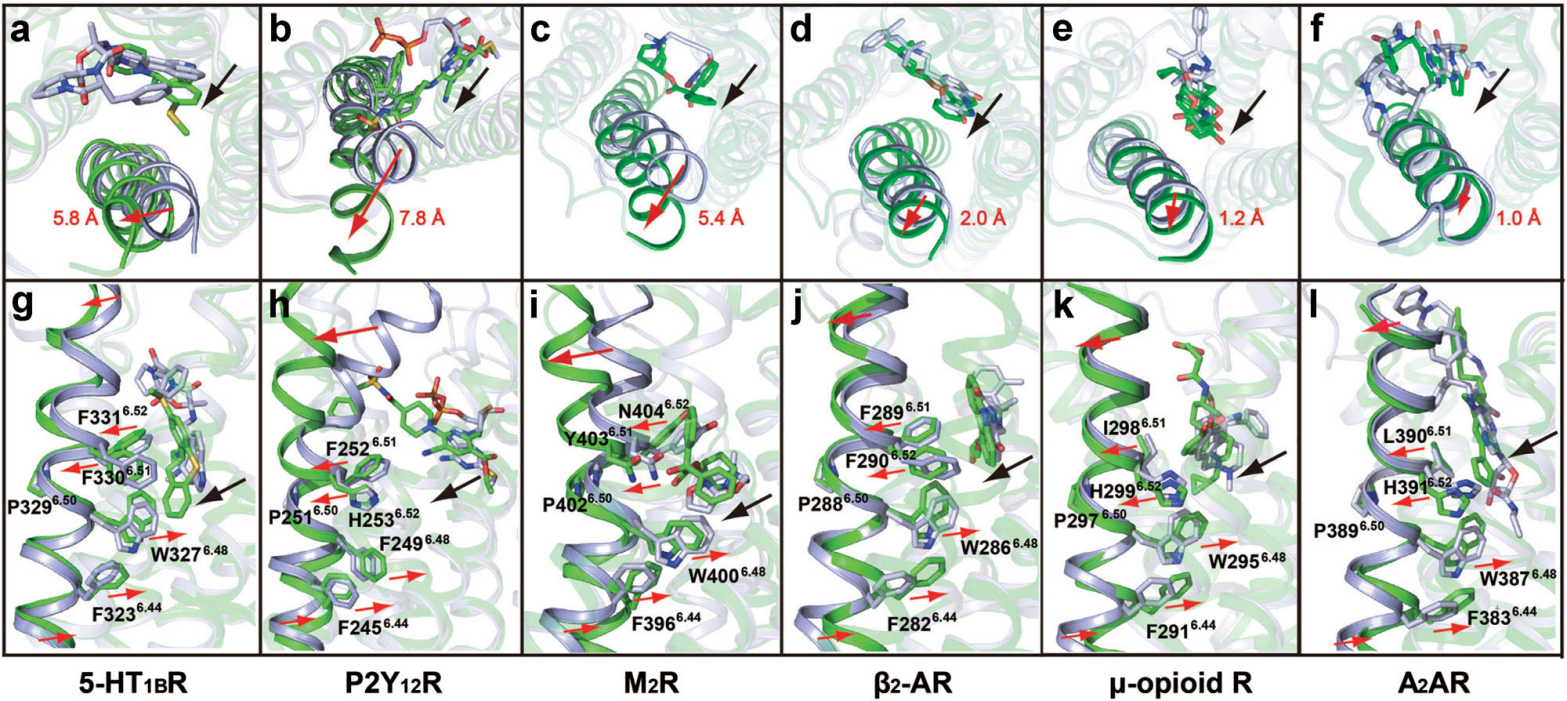
Structure alignment of TM6 and ligands of class A GPCR complexes. **a**–**f** Extracellular views of TM6 and ligands. **g**–**l** Side views of TM6 and ligands. **a** and **g** 5-HT_1B_R in complex with inverse agonist MT vs. agonist ERG (PDB code: 4IAR), **b** and **h** P2Y_12_R in complex with antagonist AZD1283 (PDB code: 4NTJ) vs. agonist 2MeSADP (PDB code: 4PXZ), **c** and **i** M_2_R in complex with inverse agonist QNB (PDB code: 3UON) vs. agonist iperoxo (PDB code: 4MQT), **d** and **j** β_2_-AR in complex with inverse agonist carazolol (PDB code: 2RH1) vs. agonist BI-167107 (PDB code: 3SN6), **e** and **k** μ-opioid R in complex with antagonist β-FNA (PDB code: 4DKL) vs. agonist BU72 (PDB code: 5C1M), **f** and **l** A_2_AR in complex with antagonist ZM241385 (PDB code: 4EIY) vs. agonist UK-432097 (PDB code: 3QAK). Red arrows indicate the movements of TM6 and the side chains of highly conserved residues at positions 6.44, 6.48, 6.51, and 6.52, and black arrows show the shifts of inverse agonists/antagonists compared with agonists in the ligand binding pockets. All GPCRs in inactive conformation are shown in green, while those in active conformation are displayed in light blue. Inverse agonists/antagonists are colored green, and agonists are in light blue. The shift distances of extracellular ends of TM6 between active and inactive conformations are labeled in red with residues at position 6.60 as reference point

The most significant activation-dependent conformational change in class A GPCRs is the outward movement of TM6 on the cytoplasmic side. Conversely, inverse agonist/antagonist-bound GPCR structures are characterized by a corresponding inward movement of TM6 at the cytoplasmic side. The crystal structure of the 5-HT_1B_R/MT complex provides a transition conformation between active and inactive states that displays an outward movement of TM6 at the extracellular side and this conformation appears to be a common structural feature shared by several other inverse agonist/antagonist-bound class A GPCR structures as depicted in Fig. 5.

In the 5-HT_1B_R/MT complex, MT occupies the same orthosteric pocket as agonist ERG in its complex structure. However, MT binds to the pocket, with the dihydrodibenzo thiepine ring rotated and shifted towards TM6, pushing away residues W327^6.48^, F330^6.51^, and F331^6.52^ on this helix, leading to the outward movement of the extracellular end of TM6 away from the TMD core (Fig. 5a, g). A larger magnitude movement at the extracellular end of TM6 can be achieved when MT adopts an energy-favorable conformation after removing OB1 fusion protein in all four simulations (Figure S3D). Besides the ligand geometry, the bend of the methylsulfanyl group toward TM6 also contributes to the outward shift of the extracellular end of TM6 (Figures S7B and S7E).

The binding of MT induces the conversion of 5-HT_1B_R from the ground state to the inactive state through its interaction with residues W327^6.48^, F330^6.51^, and F331^6.52^ of TM6. As a large number of the residues of the receptor core and ligand binding pocket are highly conserved among class A GPCRs, we asked whether this is a common inactivation mechanism. We inspected all class A GPCRs that have both agonist- and inverse agonist/antagonist-bound structures available in the Protein Data Bank (Fig. 5). Several common structural features are observed: (i) the shifts of the ring-like groups located at the bottom of the pocket from each inverse agonist/antagonist towards TM6 relative to agonist, regardless of their size, polarity, and configuration (black arrows in Fig. 5); (ii) the outward movement of the extracellular end of TM6 that occurs in all inverse agonist/antagonists-bound structures relative to that of agonists-bound ones; and (iii) the residues corresponding to W^6.48^, F^6.51^, and F^6.52^ are spatially close relative to the corresponding inverse agonist/antagonist^47–50^, and are rotated and shifted towards TM6 compared with their positions in the agonist-bound structures. These structural features provide evidence for a putative inactivation mechanism for class A GPCRs in which the bottom groups of inverse agonists/antagonists in the binding pocket rotate and shift towards TM6, inducing an outward displacement of the extracellular segment of TM6 due to steric restraints between the inverse agonist/antagonist and residue W^6.48^ as well as those at positions of 6.51 and 6.52.

**Fig. 6:**
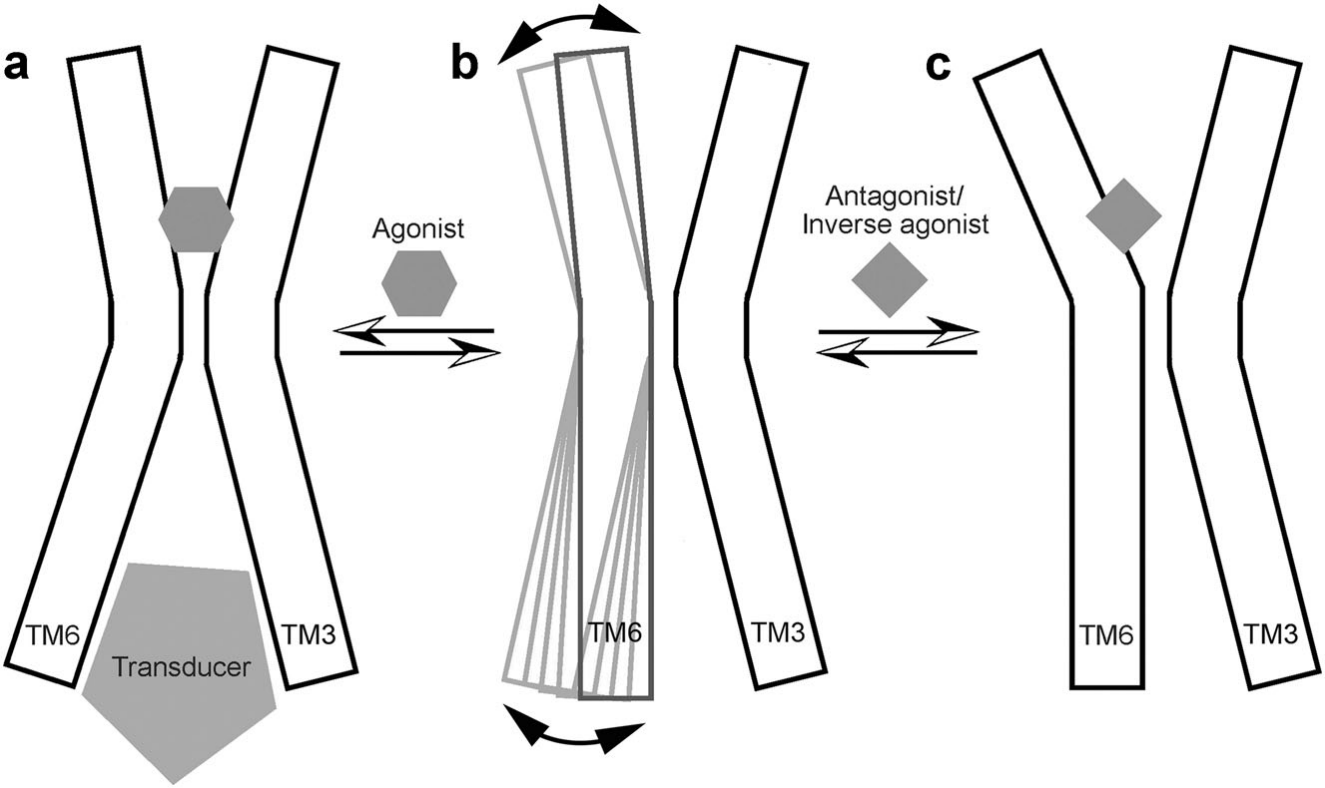
Schematic illustration of a putative mechanism of activation/inactivation of 5-HT_1B_R and other class A GPCRs. **a** GPCRs are activated by agonist binding and transduce extracellular signals to downstream pathways through recruiting effectors. Agonist is shown as grey hexagon, and transducer as grey pentagon. **b** GPCRs in ground state. **c** GPCRs inhibited by binding to antagonist or inverse agonist. Antagonist/inverse agonist is shown as grey square

A “toggle switch” model, previously proposed to explain GPCR activation, can also elucidate the outward rigid-body movement of the extracellular side of TM6 and the corresponding inward shift of the cytoplasmic side of this helix as an structural basis for 5-HT receptor inactivation^51^. Consistently, our structural and MD data demonstrate that the extracellular and intracellular segments of this helix undergo seesaw-like motion in opposite directions when the receptor binds to the inverse agonist (Fig. 6). We therefore propose that the rotation and shift of inverse agonist/antagonists towards TM6 and subsequent movement of TM6 may represent an inactivation mechanism of 5-HT receptors and some other class A GPCRs, i.e., inverse agonist/antagonist binding induces an inactive conformation of receptors through interacting with W^6.48^ and its C-terminal residues at positions 6.51 and 6.52 on TM6, leading to outward movement of the extracellular end and coupled inward shift of the intracellular end of TM6. The conserved residue W^6.48^ is known as “toggle switch” which is postulated to be the initial step in GPCR activation^51, 52^. Interestingly, the antagonist AZD1283 binds to P2Y_12_R in a distinct manner with the benzylsulphonyl group directed toward TM5, inducing the largest outward shift of the extracellular portion of the TM6 (Fig. 5b, h). Despite this receptor-specific difference, the outward movement of TM6 at the extracellular side is a common characteristic in known inverse agonist-/antagonist-bound class A GPCR structures (Fig. 5)^53^.

In this paper, we report a crystal structure of 5-HT_1B_ receptor in complex with its inverse agonist MT solved with an engineered OB1 fusion partner. The 5-HT_1B_R/MT complex structure represents an intermediate state that is prone to transition into an inactive conformation. In this complex structure, MT is deeply inserted in the orthosteric pocket in the helical bundle of the receptor. The binding of MT in the ligand binding pocket induces the outward movement of the extracellular segment of TM6 away from the helical core, initiating a cascade of conformational changes, including an inward shift of the cytoplasmic side of TM6 to block the receptor from recruiting signaling effectors, thereby holding the receptor in an inactive state. The structural features of the inverse agonist bound 5-HT_1B_R are consistent with our MD analysis and many published structural and functional studies, and provide a putative inactivation mechanism of 5-HT_1B_ receptor and some other class A GPCRs, characteristic of a “seesaw-like” swing of TM6, which moves the extracellular and intracellular ends of this helix in opposite directions.

## Materials and methods

### Generation of 5-HT_1B_R-OB1 fusion constructs

Human wide-type 5-HT_1B_R (UniProtKB ID: P28222) DNA was codon optimized and synthesized by Genewiz for insect cell expression, and OB1 (optimization variant 1 of BRIL) was codon optimized and synthesized by Genewiz for bacterial expression. The sequence of 5-HT_1B_R (S34–S390) with the point mutation L138^3.41^W^54^, which was introduced to increase the thermostability, was amplified using Phanta Super-Fidelity DNA Polymerase (Vazyme Biotech) and was subcloned into a modified pFastBac baculovirus expression vector (Invitrogen) containing an expression cassette for a hemagglutinin (HA) signal sequence, a FLAG tag, a hexa-histidine tag and a TEV protease recognition site at the N terminus. The ICL3 loop of 5-HT_1B_R (L240–M305) was replaced by OB1. The 33 N-terminal (extracellular) amino acids of 5-HT_1B_R were omitted to remove the disordered N terminus and all glycosylation sites. All plasmids were verified by sequencing with pFastBac-F and pFastBac-R primers.

### Expression and purification of 5-HT_1B_R protein for crystallization

High-titer recombinant baculovirus (>10^9^ viral particles per ml) was obtained using the Bac-to-Bac Baculovirus Expression System (Invitrogen) as previously described^55^. *Spodoptera frugiperda* (Sf9) cells at a density of 2–3 × 10^6^ cells per ml were infected with P2 virus at a multiplicity of infection (m.o.i.) of 5 in ESF921 medium (Expression System). Cells were harvested by centrifugation 48 h post-infection and stored at −80 °C until use.

Insect cell membranes were disrupted by thawing frozen cell pellets in a hypotonic buffer containing 10 mM HEPES, pH 7.5, 10 mM MgCl_2_, 20 mM KCl, and protease inhibitor cocktail (Roche). Extensive washing of the raw membranes was performed by repeated centrifugation and resuspension in the same hypotonic buffer (2 times), and then in a high osmotic buffer containing 1.0 M NaCl, 10 mM HEPES, pH 7.5, 10 mM MgCl_2_, 20 mM KCl, and protease inhibitor cocktail (3 times), thereby separating soluble and membrane associated proteins from integral transmembrane proteins.

Washed membranes were resuspended into buffer containing 20 µM of the ligand MT (Sigma), 2 mg/ml iodoacetamide, and EDTA-free complete protease inhibitor cocktail tablets, and incubated at 4 °C for 1 h before solubilization. The membranes were then solubilized in 20 mM HEPES (pH 7.5), 500 mM NaCl, 25 mM imidazole, 10% (v/v) glycerol, 0.5% (w/v) n-dodecyl-β-D-maltopyranoside (DDM, Anatrace), and 0.1% (w/v) cholesteryl hemisuccinate (CHS, Anatrace) at 4 °C for 3–4 h. The supernatant was isolated by centrifugation at 100,000 *g* for 30 min, and incubated with Ni-NTA beads (GE Healthcare) for 2 h at 4 °C. After binding, the beads was washed with 10 column volumes of Wash A Buffer (20 mM HEPES (pH 7.5), 500 mM NaCl, 25 mM imidazole, 5 µM MT, 10% (v/v) glycerol, 0.05% (w/v) DDM, 0.01% (w/v) CHS, and 10 mM ATP), followed by 5 column volumes of Wash B Buffer (20 mM HEPES (pH 7.5), 500 mM NaCl, 50 mM imidazole, 5 µM MT, 10% (v/v) glycerol, 0.05% (w/v) DDM, and 0.01% (w/v) CHS). The protein was then eluted in 3–4 column volumes of Elution Buffer (20 mM HEPES (pH 7.5), 500 mM NaCl, 200 mM imidazole, 5 µM MT, 10% (v/v) glycerol, 0.05% (w/v) DDM, and 0.01% (w/v) CHS). It was treated overnight with His-tagged TEV protease to cleave the N-terminal His-tag and FLAG-tag. A HiTrap Desalting column (GE Healthcare) was used to remove imidazole. The His-tagged TEV protease and cleaved N-terminal fragment were removed by rebinding to Ni-NTA beads, yielding pure tag-less protein. The protein was then concentrated to 30–50 mg/ml with a 100 kDa molecular weight cut-off Centrifuge Filter (Millipore). Protein purity and monodispersity were tested by SDS–PAGE and analytical size-exclusion chromatography (aSEC). Typically, the protein purity exceeded 95%, and the aSEC profile showed a single peak, indicative of receptor monodispersity (Figures S11A and S11B).

### Lipidic cubic phase crystallization

The concentrated protein was reconstituted into a mechanical syringe mixer containing monoolein plus 10% (w/w) cholesterol (Sigma), where the protein solution: lipid mass ratio was 2:3 mixed at room temperature^56^. Crystallization experiments were carried out in 96-well glass sandwich plates (Shanghai FAstal BioTech) by a Gryphon LCP crystallization robot (Art Robbins Instruments) using 40 nl protein cubic phase overlaid with 800 nl precipitant solution. Crystallization plates were incubated at 20 °C and initial crystals appeared after 48 h in about 15 conditions based on the 48 salts screening buffer. Improved crystals were obtained in a condition consisting of 100 mM Bis-Tris (pH7.0), 155 mM ammonium phosphate monobasic, 26% PEG300, 0.5 mM GSH (L-Glutathione reduced), and 0.5 mM GSSG (L-Glutathione oxidized). Crystals grew to full size (20–50 μm in one dimension) in 6–7 days (Figures S11C and S11D) and were harvested directly from LCP matrix using MiTeGen micromounts and flash frozen in liquid nitrogen.

### Data collection, structure solution and refinement

The data set to 3.90 Å was collected from 14 crystals of about 20 μm size using a 10 μm beam of 1.000 Å wavelength and 0.1 or 0.2 s exposure time per 0.1 or 0.2° oscillation with an EIGER 16 M pixel array detector at a distance of 500 mm at the X06SA beamline of the Swiss Light Source. The diffraction data were indexed and integrated with XDS^57, 58^ and scaled with XSCALE. The structure was solved by molecular replacement performed with PHASER^59^ using the 5-HT_1B_R/ERG complex structure (PDB code: 4IAR) as initial search model. The model was then manually rebuilt in COOT^60^ and refined using the PHENIX program package^61^. The data collection and model refinement statistics are listed in Table S2.

### Cell culture and transfection

CHO-K1 cells were seeded onto 96-well poly-D-lysine or fibronectin treated cell culture plates (PerkinElmer). After overnight culture, the cells were transiently transfected with wild-type or mutant 5-HT_1B_R DNA using Lipofectamine 2000 transfection reagent (Invitrogen).

### Whole-cell binding assay

The desired mutations were introduced to wild-type human 5-HT_1B_R in the pcDNA3.1 vector (Invitrogen). The mutants were constructed by PCR-based site-directed mutagenesis. Sequences of receptor clones were confirmed by DNA sequencing.

CHO cells were harvested 24 h after transfection, washed twice, and incubated with blocking buffer (F12 supplemented with 33 mM HEPES and 0.1% bovine serum albumin (BSA), pH 7.4) for 2 h at 37 °C. For homogeneous binding, the cells were incubated in binding buffer with constant concentration of [^3^H]GR125743 (1 nM) and different concentrations of unlabeled MT (6.4 pM~500 nM) at room temperature for 3 h. Cells were washed three times with ice-cold PBS and lysed by 50 μl lysis buffer (PBS supplemented with 20 mM Tris-HCl, 1% Triton X-100, pH 7.4). The plates were subsequently counted for radioactivity (counts per minute, CPM) in a scintillation counter (MicroBeta2^TM^ Plate Counter, PerkinElmer) using a scintillation cocktail (OptiPhase SuperMix, PerkinElmer).

### System preparation and molecular dynamic simulations

All-atom atmospheric simulations were performed using the GROMACS5.0.6 software suite^62^ in the isothermal isobaric (NPT) ensemble with periodic boundary conditions and the CHARMM36 force field^63^. Chain A of the 5HT_1B_/MT crystal structure reported in this manuscript (PDB: 5V54) was prepared for simulation by removing the OB1 fusion and aligned for membrane insertion using the orientations of proteins in membranes database^64^. Missing residues in ECL2 (192–196) and 3 (340–343) were modeled sequentially and subjected to 1000 rounds each of very slow loop refinement with loop scores assessed by DOPE scoring using Modeller9.13^65^. To prevent unwanted charge interactions between discontinuous free ends of TM5 and TM6, the receptor was split into two chains comprising TM1–5 and TM6-H8 respectfully as to apply neutral capping groups during GROMACS topology generation. To maintain the crystallographic polar interaction between the tertiary amine group on the piperazine ring distal to the chiral carbon and D129^3.32^, MT was protonated for a (+1) total charge and parameters were generated using the SwissParam server^66^. Optimal hydrogen bonding networks and side chain protonation states for 5-HT_1B_R were determined at pH 7.0 by PROPKA^67^ included in Schrödinger Release 2016-1. The resulting 5-HT_1B_R/MT complex was capped with neutral acetyl and methylamine groups and embedded into a palmitoyl-oleoyl-phosphatidylcholine (POPC) lipid bilayer solvated in a 73 × 73 × 89 Å box of TIP3P waters with 0.150 mM NaCl (neutralized by removing 8 sodium ions; approximately 44,000 atoms in total).

Prior to production simulations, 50,000 steps of steepest descent energy minimization was followed by equilibration in the canonical (NVT) and NPT ensembles for 10 and 50 ns respectively, with positional restraints (1000 kJ mol^−1^ nm^−2^) placed on backbone atoms. Temperature was maintained at 310 K using the v-rescale method with a coupling time of 0.1 ps and pressure was maintained at 1 bar using the Berendsen barostat with a coupling time (*t*_p_) of 1.0 ps and compressibility of 4.5 × 10^−5^ bar^−1^. Four independent 500 ns production simulations were performed for a combined 2 μs of simulation. To monitor volume of the cytoplasmic pocket over the course of simulation, trajectories were aligned based on backbone atoms of TM1–7 using MDtraj 1.7.2^68^ and analyzed using the Epock command line tool^69^. The cytoplasmic pocket was defined by an include_sphere (*r* = 9.0 Å) slightly below the “NPxxY” tyrosine hydroxyl group and a contigous_sphere (*r* = 5.0 Å) placed at the same location with a grid_spacing of 0.4 Å and contiguous_cut-off of 2.0 Å. Prior to volume calculation, the resulting pocket was visualized in VMD 1.9.2^70^.

### Density functional theory ligand geometry optimization

From the crystal structure, MT was extracted and protonated at using UCSF Chimera^71^. Geometry optimization was performed with ORCA4^72^ at the DFT level of theory using the B3LYP functional in conjunction with the RIJCOSX approximation extended basis set and the def2/J auxiliary basis set^73, 74^. Both geometry and frequency calculations were performed at all stationary points over 20 iteratins.

### Accession codes

The atomic coordinates and structure factors for 5-HT_1B_R/MT have been deposited in the Protein Data Bank, under the accession codes 5V54.

## Electronic supplementary material

Supplemental figure legends

Table S1

Table S2

Table S3

Figure S1

Figure S2

Figure S3

Figure S4

Figure S5

Figure S6

Figure S7

Figure S8

Figure S9

Figure S10

Figure S11
