# Supplementary material for: Crystal structure of the human 5-HT1B serotonin receptor bound to an inverse agonist
Source: Cell Discov. 2018 Mar 13;4:12. doi: 10.1038/s41421-018-0009-2 (PMC5847559; doi:10.1038/s41421-018-0009-2)
Supplement: Supplementary file 1 — Supplemental figure legends [file 41421_2018_9_MOESM1_ESM.docx]

**Supplementary Tables**

**Table S1**. **Radioligand competition assays of wild type and OB1 fusion constructs of 5-HT_1B_R (5-HT_1B_R-OB1).** OB1 fusion retains the binding of MT. Data represent mean pKi ± SEM for competition binding experiments using [^3^H]GR125743 as radioligand. Experiments were performed in triplicate.

**Table S2.** **Data collection and structure refinement statistics of 5-HT_1B_R/MT crystal structure.**

**Table S3**. **MT radioligand competition of 5-HT_1B_R ligand binding pocket mutants.** ND (not determined) indicates that mutations abolished binding of radioligand. Data represent mean pKi ± SEM for competition binding experiments using [^3^H]GR125743 as radioligand. Experiments were performed in triplicate.

**Supplementary Figures**

**Figure S1. 2Fo-Fc electron density map (1 σ) of the two molecules of 5-HT_1B_R/MT complex in an asymmetric unit of the C2 crystal form.** Left, molecule A; Right, molecule B.

**Figure S2. Intramolecular polar interactions within OB1 compared to those within BRIL.** (**A) and (B**), Intramolecular polar interactions from the OB1 chain B of OB1-fused 5-HT_1B_R/MT structure. (**C) and (D**), Intramolecular polar interactions within BRIL (PDB code 1M6T). OB1 is colored in green, and BRIL in orange. The polar interactions are depicted as blue dotted lines with distances labeled.

**Figure S3.** **Molecular dynamic simulations of 5-HT_1B_R/MT complex.** **(A)** A significant structural deformation (black arrow) can be observed at the linking region between 5-HT_1B_R and OB1 fusion partner in 70 ns MD. **(B)** Upon removal of the OB1, the 5-HT_1B_R cytoplasmic pocket volume significantly decreases for all simulations compared to the crystal structure. **(C-E)** Superposition of 5-HT_1B_R/MT structures in four independent 500 ns MD (grey, orange, blue and green) and its crystal structure (white). Removal of BRIL allows for a 6 Å inward movement of cytoplasmic portion of 5-HT_1B_R/MT **(D and E)**, adopting a conformation similar to that of other inactive GPCR structures, and a pronounced 3 Å outward movement of the TM6 extracellular end. **(C)**, side view; **(D)**, side view of TM6; and **(E)**, extracellular view.

**Figure S4.** **The conserved structural rearrangement of residues in the 5-HT_1B_R/MT structure.** The conformation of conserved residues of 5-HT_1B_R/MT (green) is compared with those of 5-HT_1B_R/ERG (agonist, PDB code: 4IAR, light blue), β_2_-AR/carazolol (inverse agonist, PDB code: 2RH1, magenta) and β_2_-AR/BI-167107 (agonist, PDB code: 3SN6, yellow). Side chains of conserved residues are shown as sticks. Red arrows indicate the rearrangement from active conformations to inactive conformations.

**Figure S5.** **Molecular dynamic simulation of micro switch “DRY”. (A)** The distances between D146^3.49^-R161^ICL2^ and D146^3.49^-R147^3.50^ in four independent 500 ns MD. **(B)** Micro switch “DRY” in four MD simulations. Formation of the “DRY” motif was observed only once in simulation 4 and required displacement of D146^3.49^-R161^ICL2^ and unfolding of the ICL2 helix. D146^3.49^ forms a salt bridge with R161^ICL2^ within ICl2 precluding its possibility to form a bond with R147^3.50^.

**Figure S6. Fo-Fc omit map (3 σ) of MT in the ligand binding pocket of 5-HT_1B_R (molecule A)**. Labeled are surrounding residues of the ligand binding pocket of the receptor.

**Figure S7.** **Molecular dynamic simulations of MT. (A)** Quantum Mechanics (QM) geometry optimization of MT reduces the ligand's internal energy by 43.7603 kcal/mol compared to the crystal structure. **(B)** The methylsulfanyl group of MT in QM optimized conformation bends toward the TM6 (dotted arrow) with a smaller bending angle (right), and push the extracellular portion of TM6 away from its crystal pose (left). **(C)-(E)** Removal of OB1 allows for MT to deviate from the crystal structure **(C)** and move deeper into the pocket than the crystal structure **(D and E)**.

**Figure S8: Sequence alignment of all human 5-HT receptors.** Helices are shown schematically above the sequences. Residues in the ERG binding pocket are highlighted by stars, and the ones in MT binding pocket are labeled by triangles below the sequences (red for orthosteric binding pocket and blue for extended binding pocket).

**Figure S9. Sequence alignment of MT binding pocket residues of all human 5-HT receptors.** The position of residues in the orthosteric binding pocket are labeled in red, while ones in the extended binding pocket are shown in blue.

**Figure S10.** **Binding mode comparison of MT and other 5-HT receptor ligands. (A)** The superposition of MT (green), ERG (light blue) and LSD (cyan) in 5-HT_1B_R/MT, 5-HT_1B_R/ERG (PDB code: 4IAR) and 5-HT_2B_R/LSD (PDB code: 4NTJ) binding pockets, respectively. MT inserts into the pocket 2.0 Å deeper than ERG, and 4.0 Å deeper than LSD. Polar interactions between 5-HT receptors and MT **(B)**, ERG **(C)** and LSD **(D)** shown as red dashed lines. Residues involved in polar interactions are labeled. Side chains of these polar residues are shown as sticks.

**Figure S11. Purification and crystallization of OB1-fused 5-HT_1B_R in complex with inverse agonist MT.** (**A**) SEC profile of the 5-HT_1B_/MT complex. (**B**) SDS-PAGE of OB1-fused 5-HT_1B_R. (**C**) Representative bright field (left) and birefringent (right) images of 5-HT_1B_R/MT complex crystals.
