# Supplementary material for: Crystal structure of the human 5-HT1B serotonin receptor bound to an inverse agonist
Source: Cell Discov. 2018 Mar 13;4:12. doi: 10.1038/s41421-018-0009-2 (PMC5847559; doi:10.1038/s41421-018-0009-2)
Supplement: Supplementary file 2 — Table S1 [file 41421_2018_9_MOESM2_ESM.docx]

Table S1. Radioligand competition assays on wild type and OB1 fusion constructs of 5-HT_1B_ receptor (5-HT_1B_-OB1). OB1 fusion retains the binding of MT. Data represent mean pKi (pKi ± SEM) for competition binding experiments using [^3^H]GR125743 as radioligand. Experiments were performed in triplicate.

|  | 5-HT_1B_ WT | 5-HT_1B_-OB1 |
| --- | --- | --- |
|  | (pKi±SEM) | (pEC50±SEM) |
| MT | 8.19±0.29 | 8.42±0.14 |
