# Supplementary material for: Crystal structure of the human 5-HT1B serotonin receptor bound to an inverse agonist
Source: Cell Discov. 2018 Mar 13;4:12. doi: 10.1038/s41421-018-0009-2 (PMC5847559; doi:10.1038/s41421-018-0009-2)
Supplement: Supplementary file 3 — Table S2 [file 41421_2018_9_MOESM3_ESM.docx]

| Data collection | |
| --- | --- |
| Space group | C2 |
| Resolution range, Å | 45.5－3.9 (4.0-3.9)* |
| Cell parameters, Å, ° | a=235.2, b=48.8, c=139.2  β=124.07 |
| Total/Unique reflections | 91899/12131 |
| Completeness, % | 97.3 (83.4) |
| Mean I/σ | 4.61 (0.74) |
| Multiplicity | 7.4 (2.0) |
| Rmerge | 0.23 (1.08) |
| CC1/2 | 99.4 (58.5) |
| Refinement | |
| Resolution, Å | 45.5－3.9 |
| No. reflections | 11996 |
| No. residues | 767 |
| No. solvent molecules | 0 |
| No. non-H atoms | 6180 |
| Rcryst % | 27.4 |
| Rfree % | 28.9 |
| rmsd bonds, Å | 0.006 |
| rmsd angles, ° | 1.21 |
| Wilson B factor, Å2 | 152.0 |
| B factor of protein, Å2 | 201.4 |
| B factor of ligand, Å2 | 176.5 |
| Ramachandran favored/outlier % | 98.6/0.0 |
| Molprobity score | 1.3 |

Table S2. Data collection and structure refinement statistics of 5-HT_1B_R/MT crystal structure

*Values in parentheses are for the highest resolution shell.
