# Supplementary material for: Crystal structure of the human 5-HT1B serotonin receptor bound to an inverse agonist
Source: Cell Discov. 2018 Mar 13;4:12. doi: 10.1038/s41421-018-0009-2 (PMC5847559; doi:10.1038/s41421-018-0009-2)
Supplement: Supplementary file 4 — Table S3 [file 41421_2018_9_MOESM4_ESM.docx]

Table S3. Radioligand competition of MT on ligand binding pocket mutants of the 5-HT_1B_ receptor. ND (not determined) indicates that mutations abolished binding of radioligand. Data represent mean pKi (pKi ± SEM) for competition binding experiments using [^3^H]GR125743 as radioligand. Experiments were performed in triplicate.

|  | Binding Assay | Relative Affinity  Decrease to WT* |
| --- | --- | --- |
|  | pKi±SEM |  |
| WT | 8.19±0.29 | 1.00 |
| W125^3.28^ | 8.92±0.65 | 0.18 |
| L126^3.29^ | 8.32±1.02 | 0.70 |
| D129A^3.32^ | ND | >100 |
| I130A^3.33^ | 8.52±0.17 | 0.21 |
| C133A^3.36^ | 8.61±0.21 | 0.22 |
| T134A^3.37^ | 7.39±0.25 | 11.99 |
| I137A^3.40^ | 7.57±0.11 | 5.05 |
| V201A^ECL2^ | ND | >100 |
| S212A^5.42^ | 7.73±0.14 | 4.45 |
| T213A^5.43^ | ND | >100 |
| A216S^5.46^ | 8.86±0.11 | 0.18 |
| W327A^6.48^ | ND | >100 |
| F330A^6.51^ | ND | >100 |
| F331A^6.52^ | ND | >100 |
| T355A^7.39^ | 7.2±0.08 | 11.44 |
| Y359A^7.43^ | ND | >100 |

*Relative Affinity Decrease to WT=Ki_Mutant_/Ki_WT_
