# Supplementary figures and images for: Crystal structure of the human 5-HT1B serotonin receptor bound to an inverse agonist
Source: Cell Discov. 2018 Mar 13;4:12. doi: 10.1038/s41421-018-0009-2 (PMC5847559; doi:10.1038/s41421-018-0009-2)

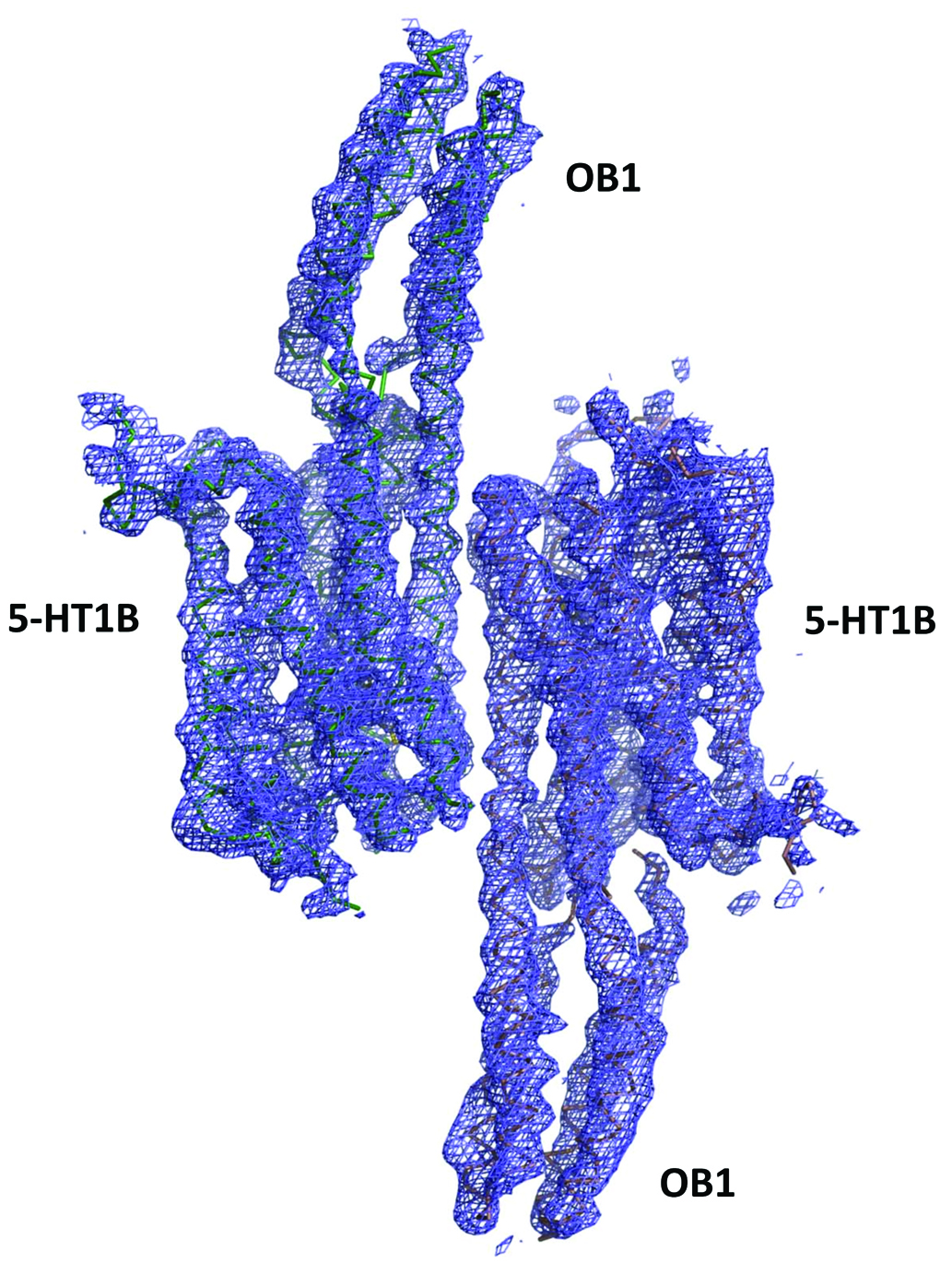

Supplement: Supplementary file 5 — Figure S1 [file 41421_2018_9_MOESM5_ESM.jpg]

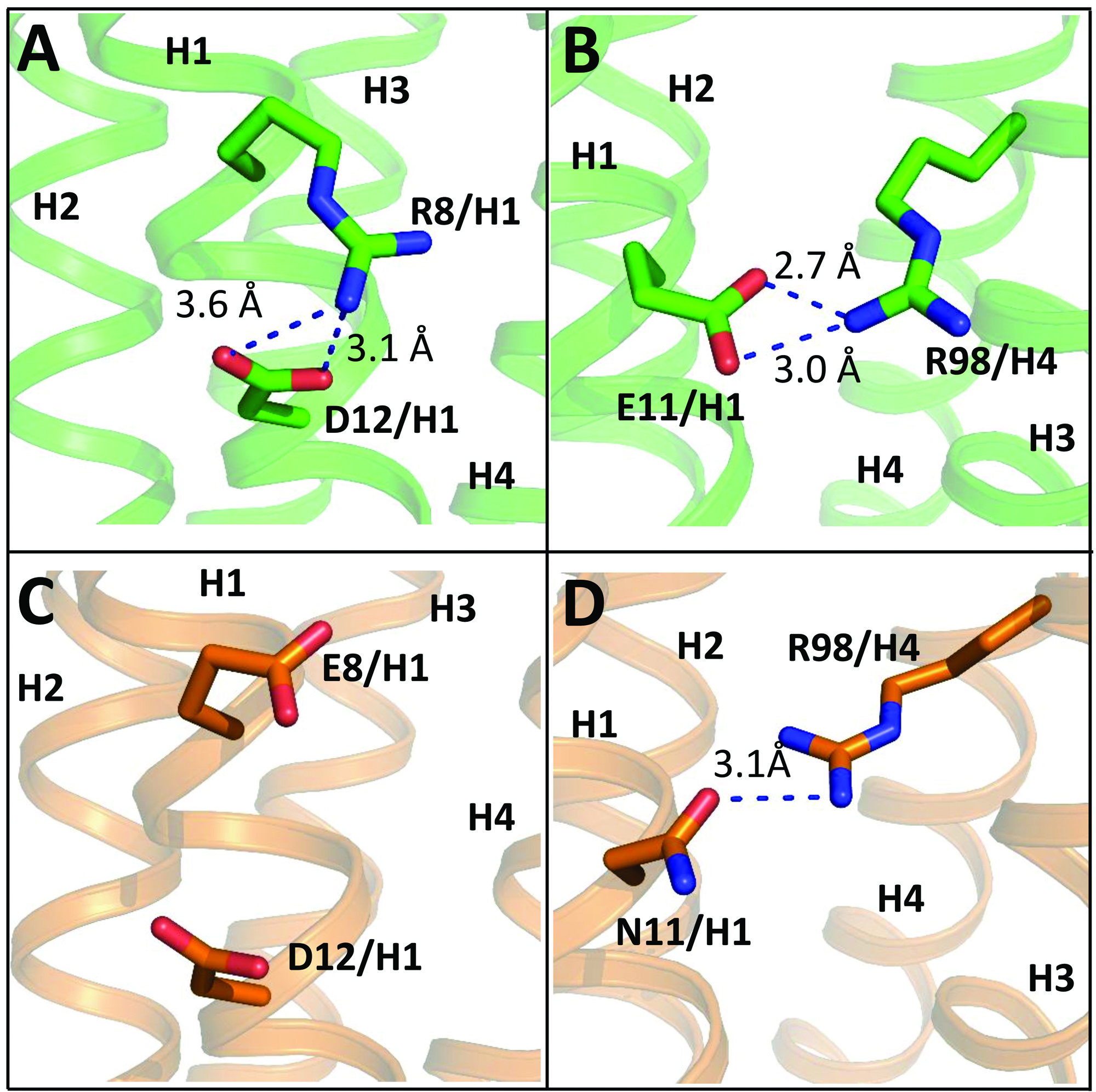

Supplement: Supplementary file 6 — Figure S2 [file 41421_2018_9_MOESM6_ESM.jpg]

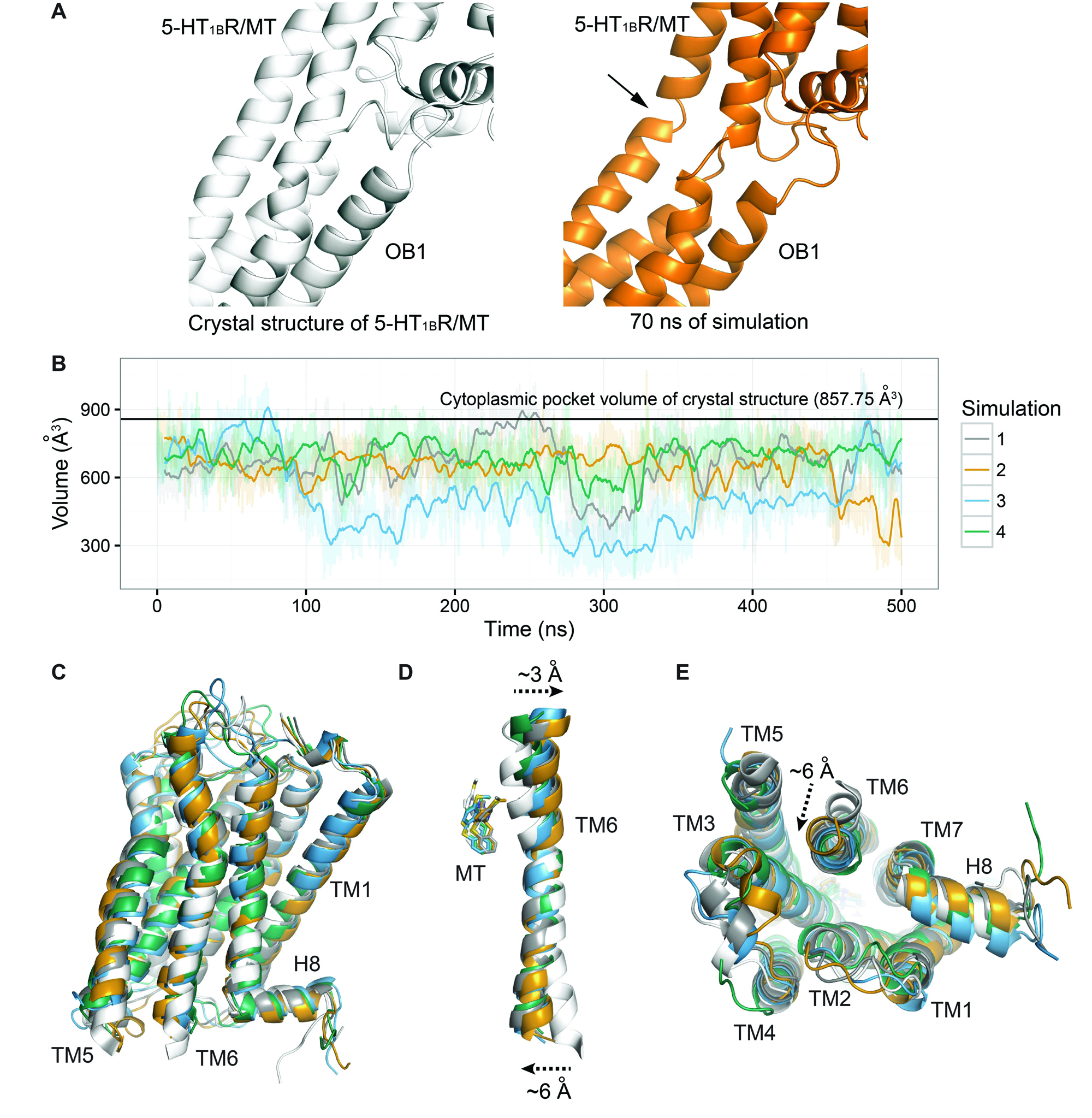

Supplement: Supplementary file 7 — Figure S3 [file 41421_2018_9_MOESM7_ESM.jpg]

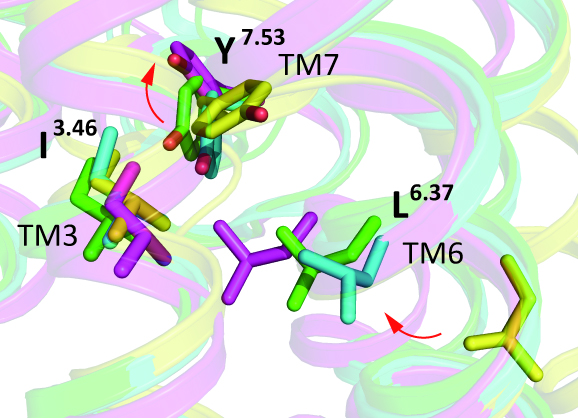

Supplement: Supplementary file 8 — Figure S4 [file 41421_2018_9_MOESM8_ESM.jpg]

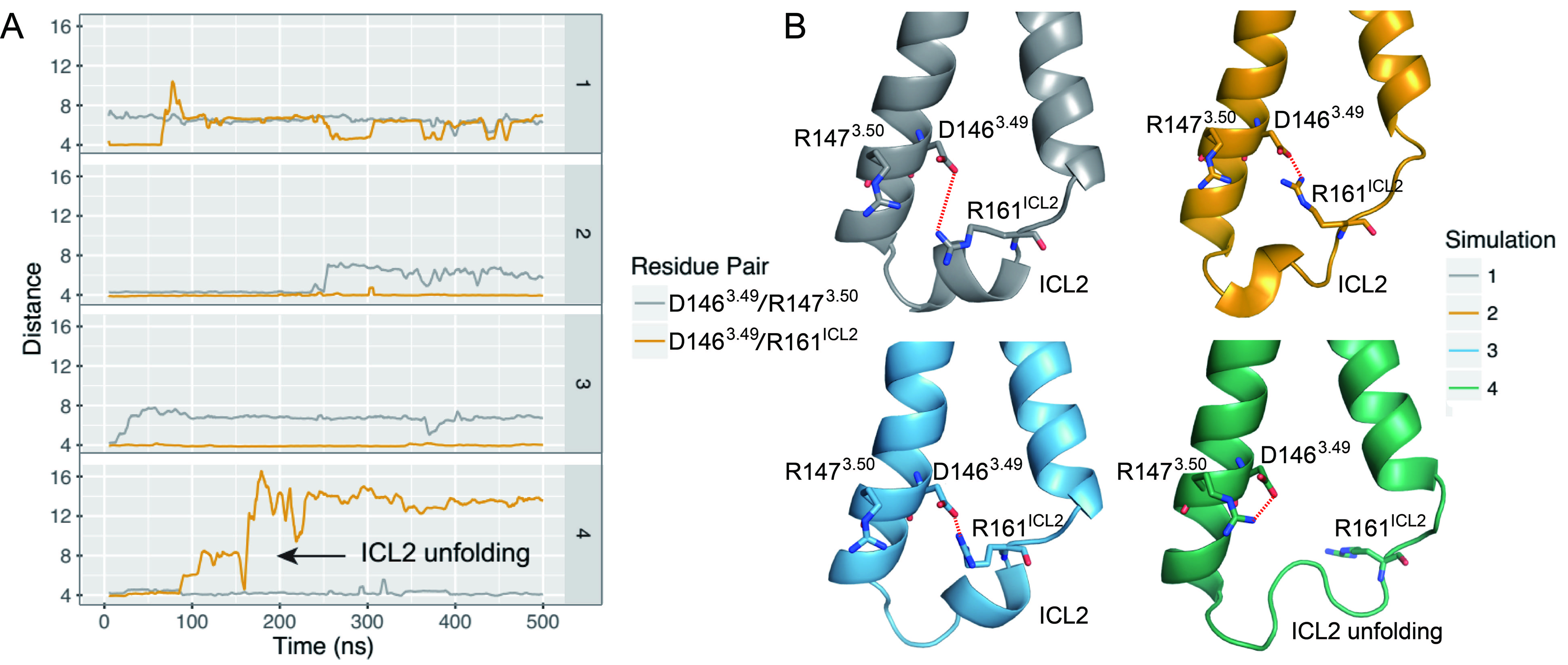

Supplement: Supplementary file 9 — Figure S5 [file 41421_2018_9_MOESM9_ESM.jpg]

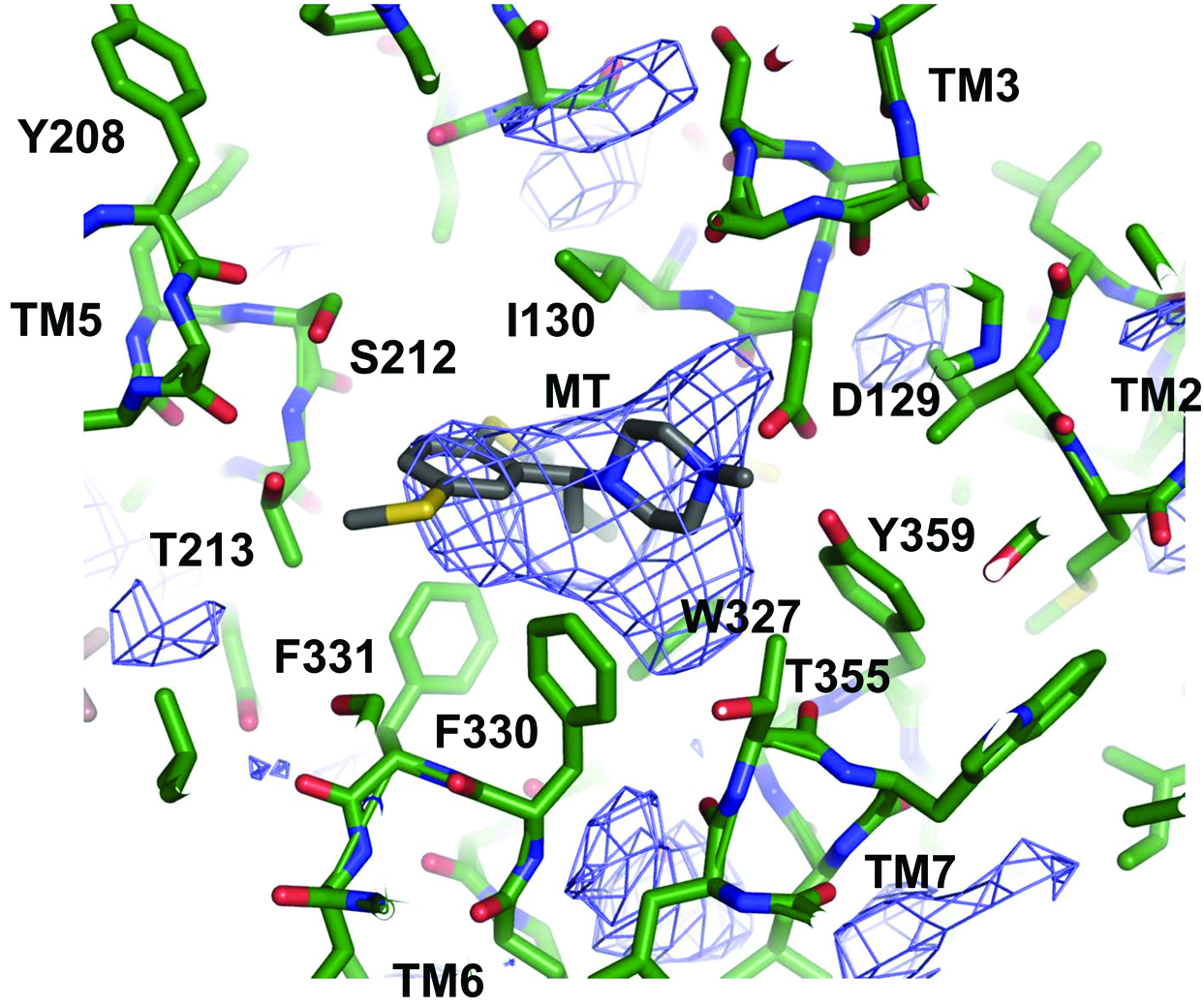

Supplement: Supplementary file 10 — Figure S6 [file 41421_2018_9_MOESM10_ESM.jpg]

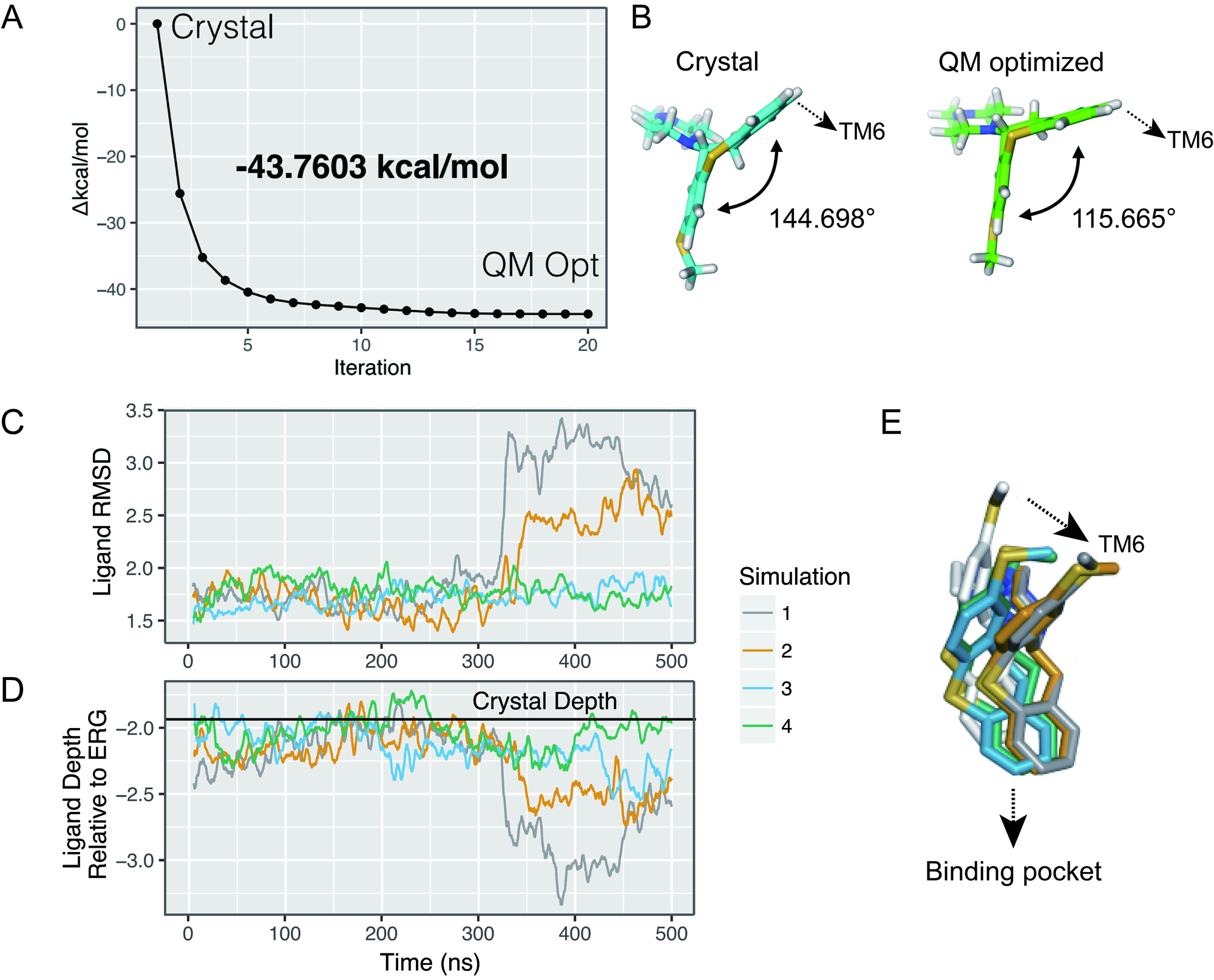

Supplement: Supplementary file 11 — Figure S7 [file 41421_2018_9_MOESM11_ESM.jpg]

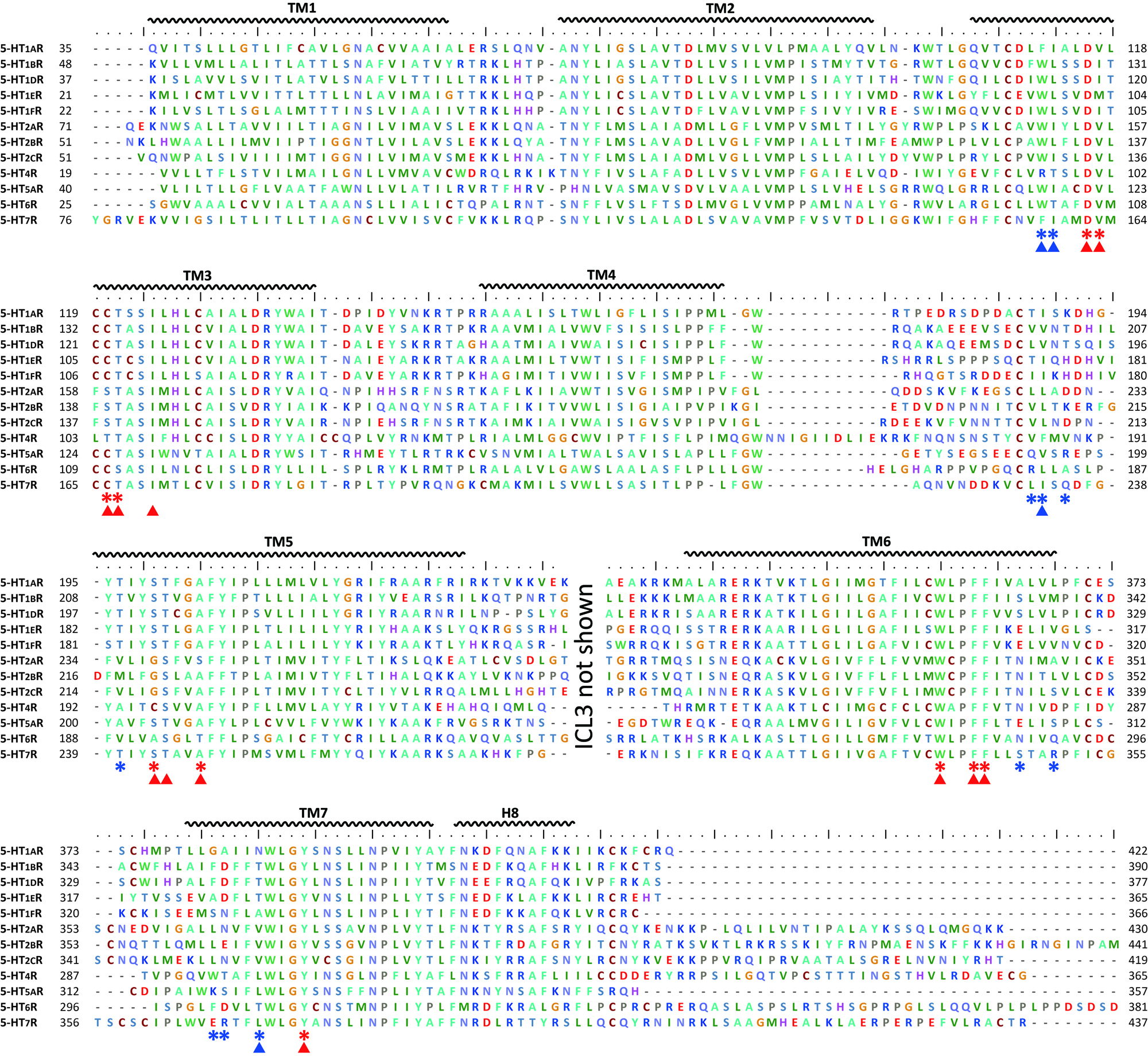

Supplement: Supplementary file 12 — Figure S8 [file 41421_2018_9_MOESM12_ESM.jpg]

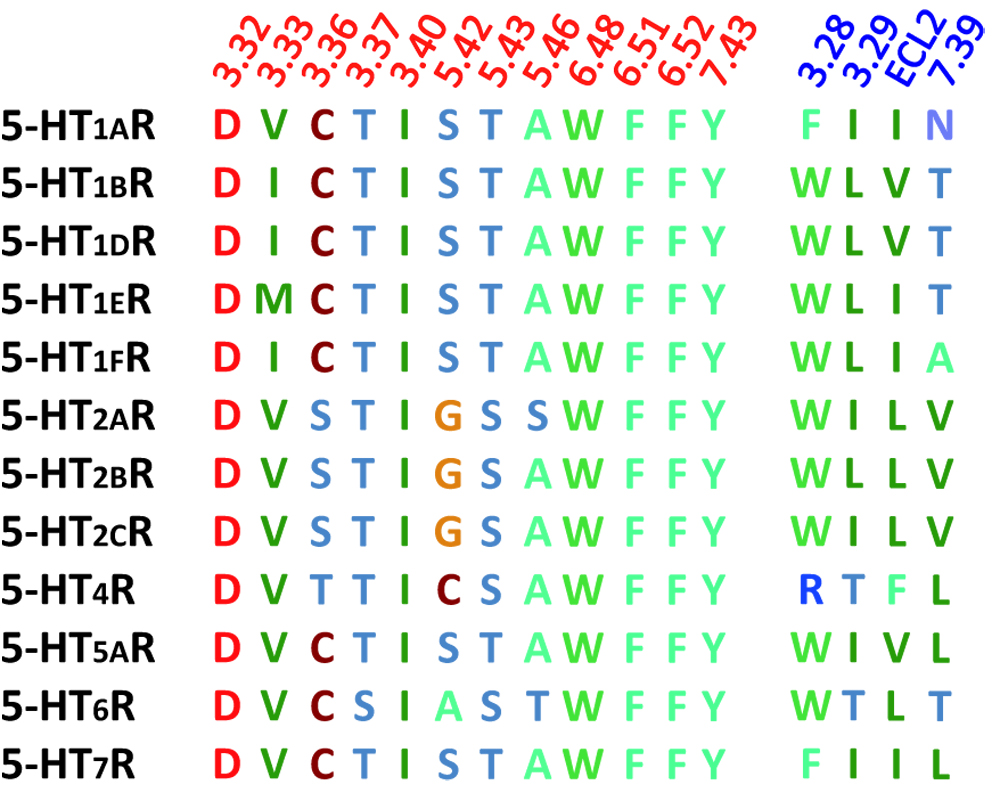

Supplement: Supplementary file 13 — Figure S9 [file 41421_2018_9_MOESM13_ESM.jpg]

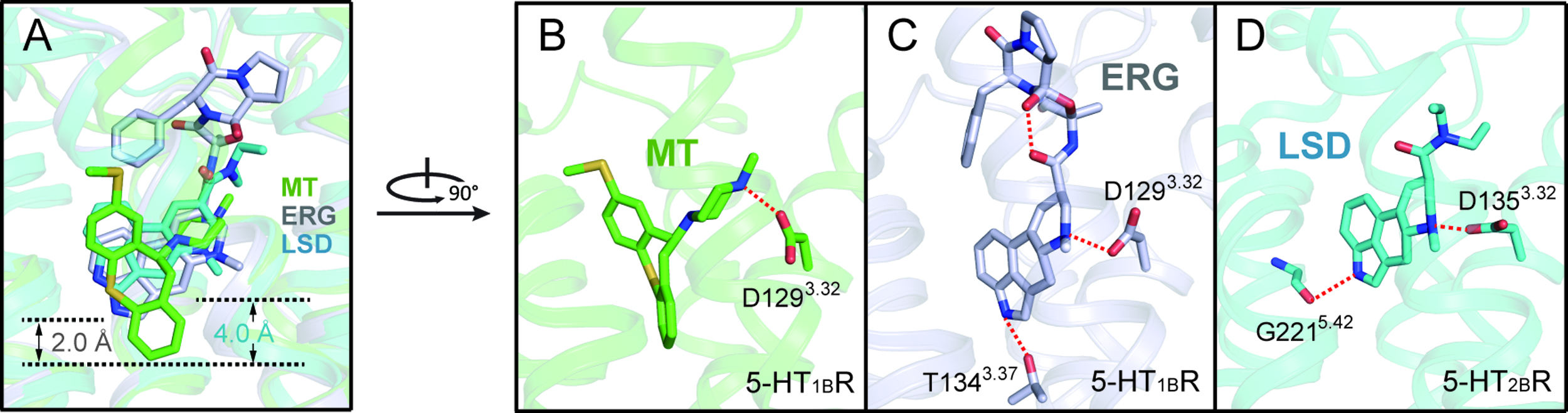

Supplement: Supplementary file 14 — Figure S10 [file 41421_2018_9_MOESM14_ESM.jpg]

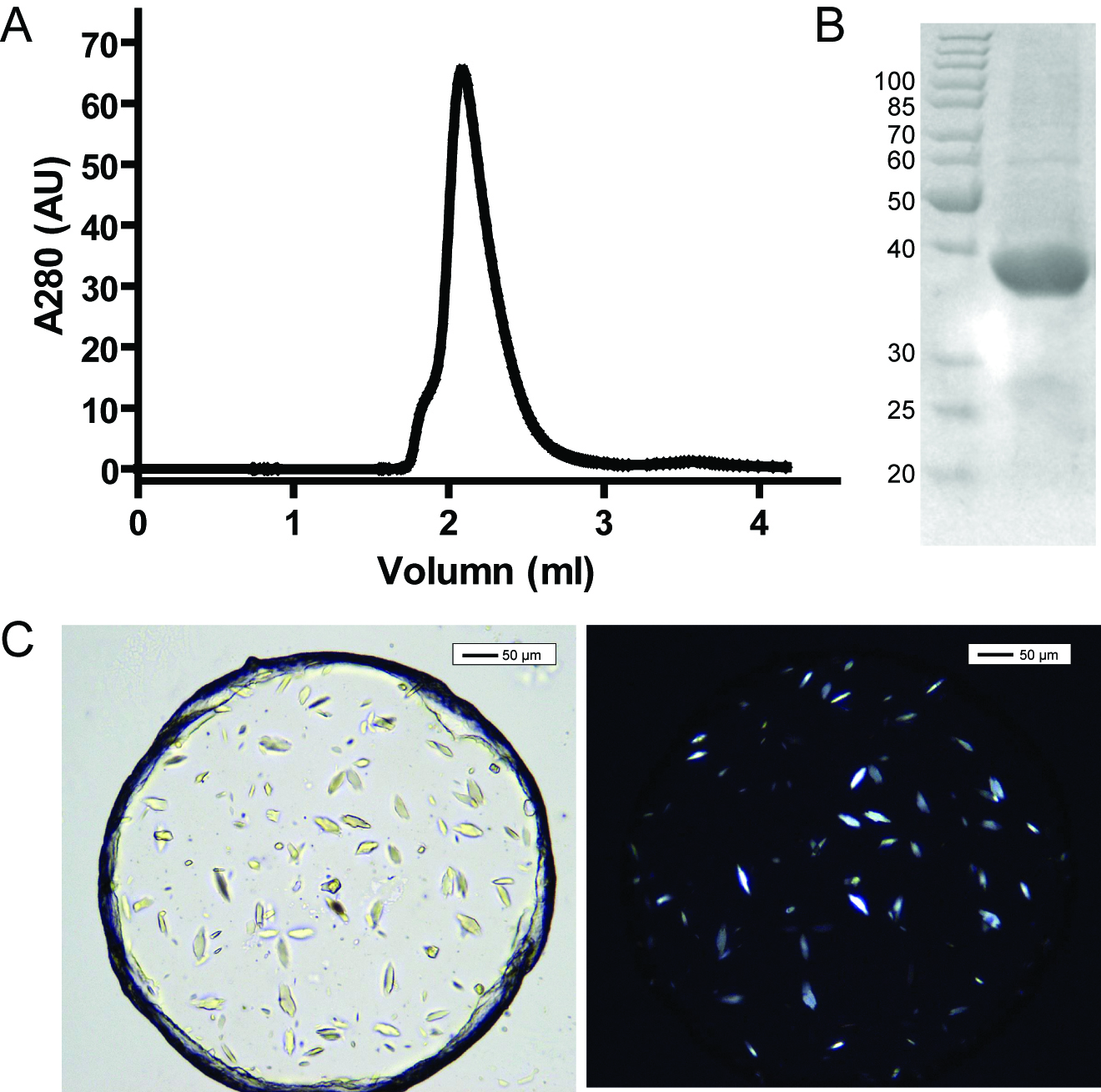

Supplement: Supplementary file 15 — Figure S11 [file 41421_2018_9_MOESM15_ESM.jpg]
